# A small-molecule inhibitor of hypoxia-inducible factor prolyl hydroxylase improves obesity, nephropathy and cardiomyopathy in obese ZSF1 rats

**DOI:** 10.1371/journal.pone.0255022

**Published:** 2021-08-02

**Authors:** Pierre E. Signore, Guangjie Guo, Zhihua Wei, Weihua Zhang, Al Lin, Ughetta del Balzo

**Affiliations:** FibroGen, Inc., San Francisco, CA, United States of America; University Medical Center Utrecht, NETHERLANDS

## Abstract

Prolyl hydroxylase (PH) enzymes control the degradation of hypoxia-inducible factor (HIF), a transcription factor known to regulate erythropoiesis, angiogenesis, glucose metabolism, cell proliferation, and apoptosis. HIF-PH inhibitors (HIF-PHIs) correct anemia in patients with renal disease and in animal models of anemia and kidney disease. However, the effects of HIF-PHIs on comorbidities associated with kidney disease remain largely unknown. We evaluated the effects of the HIF-PHI FG-2216 in obese ZSF1 (Ob-ZSF1) rats, an established model of kidney failure with metabolic syndrome. Following unilateral nephrectomy (Nx) at 8 weeks of age, rats were treated with 40 mg/kg FG-2216 or vehicle by oral gavage three times per week for up to 18 weeks. FG-2216 corrected blood hemoglobin levels and improved kidney function and histopathology in Nx-Ob-ZSF1 rats by increasing the glomerular filtration rate, decreasing proteinuria, and reducing peritubular fibrosis, tubular damage, glomerulosclerosis and mesangial expansion. FG-2216 increased renal glucose excretion and decreased body weight, fat pad weight, and serum cholesterol in Nx-Ob-ZSF1 rats. Additionally, FG-2216 corrected hypertension, improved diastolic and systolic heart function, and reduced cardiac hypertrophy and fibrosis. In conclusion, the HIF-PHI FG-2216 improved renal and cardiovascular outcomes, and reduced obesity in a rat model of kidney disease with metabolic syndrome. Thus, in addition to correcting anemia, HIF-PHIs may provide renal and cardiac protection to patients suffering from kidney disease with metabolic syndrome.

## Introduction

Chronic kidney disease (CKD) is a major challenge in public health, affecting an estimated 15% of people in the United States and more than 11% worldwide [1, 2]. CKD is associated with several comorbidities, including hypertension, heart failure, diabetes, obesity, and hyperlipidemia [3–5]. Anemia is also a common complication of renal disease because failing kidneys do not produce enough erythropoietin (EPO) to maintain normal levels of red blood cells, and the liver cannot fully compensate by secreting its own EPO [6]. The current standard therapy for anemia of renal failure is iron supplementation and, in more severe cases, administration of recombinant human EPO (rhEPO) [7, 8]. However, the latter treatment regimen induces levels of circulating rhEPO that greatly exceed the normal physiological range for endogenous EPO, which leads to an increased rate of cardiovascular events [9, 10].

A novel approach to treating anemia of CKD targets prolyl hydroxylase (PH) enzymes that are key regulators of hypoxia-inducible factors (HIFs) [11]. HIFs are transcription factors that function as heterodimers comprising an oxygen-regulated α-subunit and a constitutively expressed β-subunit. Under normoxic conditions, PH enzymes hydroxylate two conserved proline residues on HIF-α subunits. This hydroxylation promotes HIF-α binding to the von Hippel Lindau protein, resulting in proteasomal degradation of HIF-α. PH enzymes use oxygen as a co-substrate, so PH activity is suppressed under hypoxic conditions, allowing HIF-α to escape hydroxylation and degradation. HIF-α then translocates to the nucleus and forms a heterodimer with HIF-β subunit that activates the transcription of 100–200 target genes. These genes contain a hypoxia response element in their promoter region and are involved in a range of processes, including erythropoiesis, vascular tone control, angiogenesis, glucose and lipid metabolism, cell proliferation, and apoptosis [12, 13].

Since the discovery that HIF-PHs regulate erythropoiesis, several HIF-PH inhibitors (HIF-PHIs) have been developed and are currently in clinical trials [11, 14]. HIF-PHIs correct anemia in CKD patients [7, 15], as well as in animal models of anemia and kidney disease [16–19]. However, the effects of HIF-PHIs on other conditions associated with renal disease remain unknown.

The HIF-PHI FG-2216 has been shown to increase HIF-α levels in the heart, kidney and liver [20, 21]. In this study, we investigated whether, in addition to stimulating erythropoiesis, FG-2216 also protects against comorbidities associated with renal failure. To this end, we evaluated the effects of FG-2216 in obese ZSF1 rats, a model of kidney failure with metabolic syndrome that develops hypertension, renal dysfunction, hyperlipidemia, hyperglycemia, and heart failure [22, 23].

## Methods

### Animals

Eight-week-old male obese ZSF1 *Lepr*^*fa*^ *Lepr*^*cp*^ (Ob-ZSF1), lean ZSF1 (Ln-ZSF1), and Wistar-Kyoto (WKY) rats were purchased from Charles River (Saint Constant, Quebec, Canada). ZSF1 rats are generated by crossing female heterozygous lean Zucker Diabetic Fatty rats with male heterozygous lean Spontaneously Hypertensive Heart Failure (SHHF/Mcc-facp) rats [23]. Rats were housed in pairs by treatment group in the animal care facility at FibroGen, Inc. (San Francisco, CA) following the FibroGen Animal Care and Use Handbook 2011. Briefly, rats were housed in a temperature- and humidity-controlled facility with a 12-hour light/dark cycle and *ad libitum* access to water and Purina 5008 rodent diet (LabDiet, St. Louis, MO). All procedures were reviewed and approved by the Institutional Animal Care and Use Committee at FibroGen. Animals were monitored at least once a day for the duration of the study by trained vivarium staff who assessed their general appearance and spontaneous behavior.

### Study design

#### Renal function

**Fig 1:**
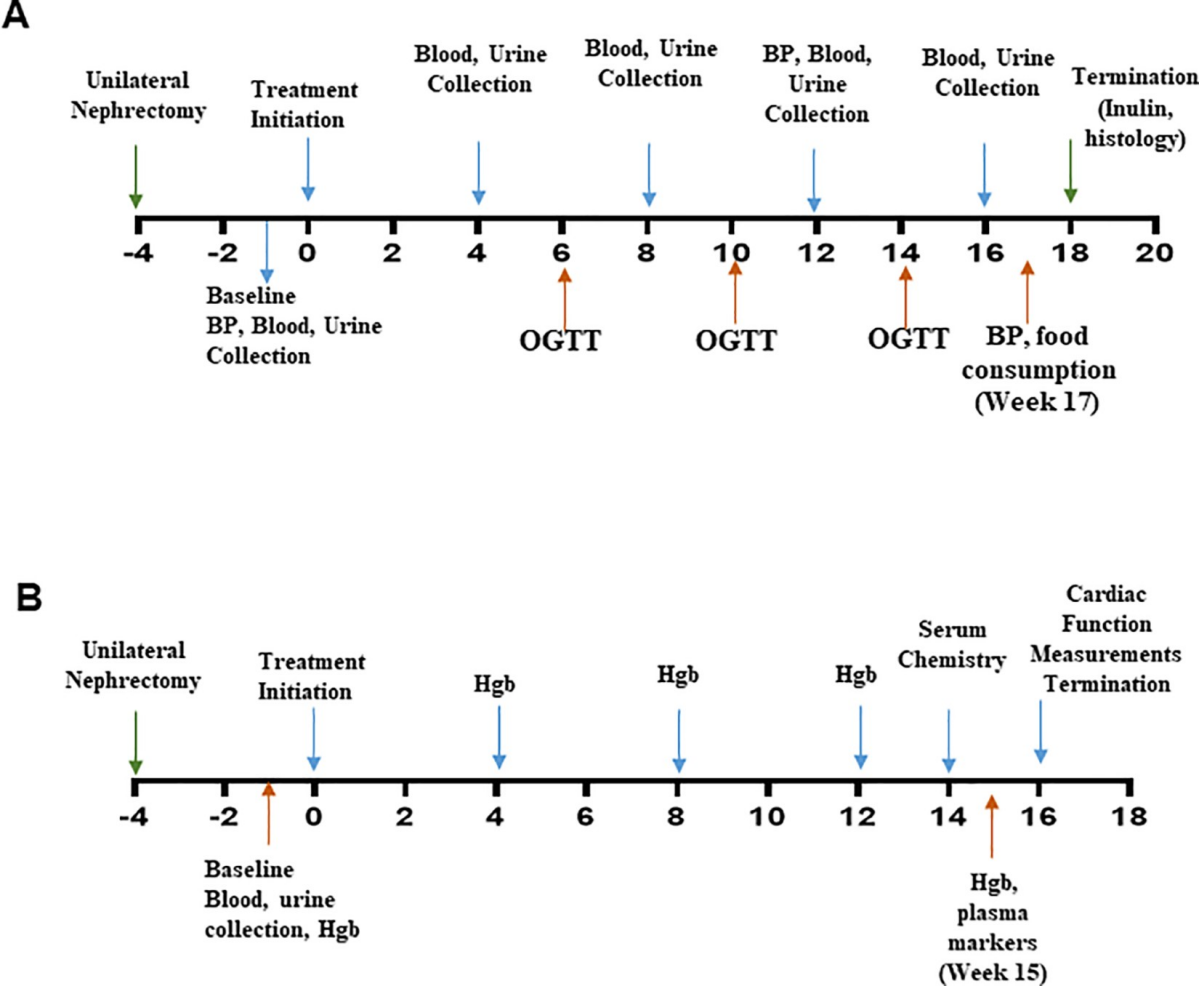
Experimental study design. Study design of renal (A) and cardiac (B) studies.

Eight-week-old Ln-ZSF1 (n = 9) and Ob-ZSF1 (n = 32) rats were allowed to acclimate for a minimum of 7 days before starting study procedures (Fig 1*A*). Ob-ZSF1 rats then underwent sterile unilateral nephrectomy (Nx) to accelerate the development of nephropathy in these animals [24]. Age-matched Ln-ZSF1 rats underwent a sham procedure. Three weeks after surgery, baseline blood pressure was measured by the tail-cuff method, blood was collected for baseline hematology and serum chemistry, and urine was collected for 24 hours for baseline urine analysis. Nx-Ob-ZSF1 rats were then assigned to one of two treatment groups (FG-2216 or Vehicle) to obtain comparable mean group values for serum levels of blood urea nitrogen (BUN) and creatinine, as well as body weight. Two Nx-Ob-ZSF1 rats and one Ln-ZSF1 sham rat with the most extreme values were excluded from the study.

Following a 3-week recovery period, Nx-Ob-ZSF1 rats were treated with FG-2216 or vehicle, and Ln-ZSF1 were treated with vehicle beginning on the Monday of the fourth week following nephrectomy, and then 3 times per week for 18 weeks. After 12 and 17 weeks of treatment, systemic blood pressure was measured 3 hours after treatment with FG-2216 or vehicle by the tail-cuff method. After 17 weeks of treatment, blood pressure was also measured 24 hours after dose administration. Every four weeks (after 4, 8, 12, and 16 weeks of treatment), blood was collected for hematology and serum chemistry approximately 6 hours after treatment, after which urine was collected for 24 hours for chemical analysis. At 16 weeks, blood samples were also collected to measure plasma markers of cardiac hypertrophy and injury with the Rat NT-proBNP Kit and the Cardiac Injury Panel 3 Rat Kit from Meso Scale Discovery (Gaithersburg, MD). On alternate weeks (6, 10, and 14 weeks), a glucose tolerance test was conducted approximately 6 hours after FG-2216 or vehicle administration. After 17 weeks of treatment, food consumption and water intake were measured over 48 hours, encompassing a dosing and a non-dosing day. Three to five days after the final treatment dose at 18 weeks, glomerular filtration rate (GFR) was determined with an inulin clearance assay under anesthesia. After completion of the inulin assay, the anesthetized animals were euthanized by thoracotomy followed by exsanguination with physiological saline perfused throughout the circulatory system via the left cardiac ventricle. Tissues were then harvested for histology and immunohistochemistry.

#### Cardiac function

Eight-week-old WKY (n = 10) and Ob-ZSF1 (n = 30) rats were allowed to acclimate for 2 weeks before starting study procedures (Fig 1*B*). Ob-ZSF1 rats underwent sterile unilateral Nx to accelerate the development of nephropathy in these animals [24]. Age-matched WKY rats underwent a sham procedure. Three weeks after surgery, blood was collected to measure baseline serum BUN, creatinine, and cholesterol, and urine was collected for 24 hours to measure baseline proteinuria. Nx-Ob-ZSF1 rats were then assigned to one of two treatment groups (FG-2216 or Vehicle) to obtain comparable mean group values for BUN and serum creatinine, as well as body weight. Two Nx-Ob-ZSF1 rats and one WKY sham rat with the most extreme values were excluded from the study.

Following a 3-week recovery period, Nx-Ob-ZSF1 rats were treated with FG-2216 or vehicle, and WKY rats were treated with vehicle beginning on the Monday of the fourth week after nephrectomy, and then 3 times per week for 16 weeks. After 14 weeks of treatment, blood was collected for serum chemistry. After 15 weeks of treatment, blood was collected to measure plasma levels of NT-proBNP (Rat NT-proBNP Kit, Meso Scale Discovery, Gaithersburg, MD). At several time points during the treatment period (4, 8, 12, and 15 weeks) and at baseline before starting treatment, a drop of blood was collected to measure blood hemoglobin concentration with a HemoPoint H2 Handheld System (Abbott Laboratories, Abbott Park, IL). After 16 weeks of treatment, 2–4 days after the last dose of FG-2216 or vehicle, left ventricular function was determined by analyzing pressure-volume loops under anesthesia. After completion of cardiac function measurement, the anesthetized animals were euthanized by thoracotomy and exsanguination with physiological saline perfused throughout the circulatory system. Their heart, lungs, kidneys, and epididymal fat pad were then harvested and weighed.

### Unilateral nephrectomy

Rats undergoing unilateral nephrectomy were anesthetized with isoflurane, and their body temperature was maintained using water-circulating heating blankets. For pain management, rats were injected subcutaneously with Buprenex (0.01 mg/kg; Reckitt Benckiser Healthcare Ltd., Slough, United Kingdom) before surgery and with bupivacaine (0.5 mg/rat; Hospira, Inc., Lake Forest, IL) along the incision site. An incision was made along the midline of the abdomen, followed by blunt dissection and ligation of the left renal pedicle to remove the kidney while leaving the adrenal gland in place. Warm (~37°C) physiological saline was administered directly in the abdomen as fluid therapy. The incision was sutured in layers. For sham animals, surgical procedures were performed as above without kidney resection. Rats were then placed in a clean cage with a warm water-circulating pad underneath and observed by the laboratory personnel until full recovery from anesthesia as indicated by normal respiratory behavior and posture/locomotion. The animals were then returned to their regular holding room with free access to food and water. Their cage was kept (half off / half on) on a warm water-circulating pad for 24 hours post-operatively to provide warmth. To manage pain after surgery, rats were given access to water jelly containing buprenorphine (2.3 mg/L; Reckitt Benckiser Healthcare Ltd., Hull, England) at the bottom of the cage. To help rats access food during the recovery period, they were floor-fed with Purina 5008 rodent diet for at least 3 days after surgery.

### FG-2216 administration

FG-2216 ([(1-Chloro-4-hydroxy-isoquinoline-3-carbonyl)-amino]-acetic acid) supplied by FibroGen, Inc. (San Francisco, CA, USA) was prepared as micronized suspensions (8 mg/mL) in water containing 0.5% carboxymethylcellulose sodium (Na-CMC) and 0.1% polysorbate 80. Vehicle was an aqueous solution containing 0.5% Na-CMC and 0.1% polysorbate 80.

Rats were treated with FG-2216 (40 mg/kg) or vehicle control by oral gavage. Each treatment dose was calculated based on the rat’s body weight recorded on the days of treatment.

### Hematology and urine analysis

Blood was collected in EDTA tubes (0.5 mL) for hematology, in BD Microtainer tubes with serum separator (0.6 mL) for serum chemistry (SRI Lab, Menlo Park, CA), and in heparin tubes (0.4 mL) for plasma NT-proBNP and cardiac-injury panel analysis. EDTA tubes were stored at 4°C until shipment for hematology analysis (SRI Lab, Menlo Park, CA) within 24 hours of collection. Blood collected in BD Microtainer tubes with serum separator sat at room temperature for 30 minutes to allow clot formation. Blood collected in heparin tubes were kept on ice during collection. Serum separator and heparin tubes were then centrifuged at 6,000 x *g* and 4°C for 5 min. Immediately after centrifugation, serum and plasma were transferred to pre-labeled Eppendorf tubes and stored at -20°C until analysis.

Following blood collection, rats were individually housed in metabolic cages to measure 24-hour urine volume and urine chemistry. While in the metabolic cages, rats were food-fasted with free access to water. Urine samples were stored at -20°C until shipped for analysis (SRI Lab, Menlo Park, CA).

### Glucose tolerance test

For oral glucose tolerance tests (OGTTs), rats were food-fasted overnight and then given their scheduled dose of FG-2216 or Vehicle the following day. Approximately 6 hours after dosing, rats were administered glucose (2 g/kg; 5 mL/kg from a 0.4 g/mL glucose solution in deionized water; EMD Millipore Corporation; Billerica, MA) by oral gavage. A drop of blood was collected from the tail vein of rats to measure blood glucose with an AlphaTRAK2 glucometer before and 30, 60, 90 and 120 minutes after glucose administration. During OGTTs, rats were kept warm with a heating pad to induce mild vasodilation of tail vessels to ease blood collection. Before blood collection, rats were given local anesthesia with a topical lidocaine (2.5%) and prilocaine (2.5%) cream (Akorn, Lake Forest, IL) applied to the tail. The tail was wiped clean before collecting blood.

### Glomerular filtration rate

Inulin clearance was used to calculate GFR. Rats were weighed and anesthetized with isoflurane, followed by cannulation of one femoral vein, one femoral artery, and the bladder. A bolus of 4% inulin (0.6 mL; MilliporeSigma Co, St. Louis, MO) was administered intravenously, followed by infusion of 4% inulin through the venous catheter with a pump at approximately 40 μL/minute. After a 45-minute equilibrium period, inulin clearance was measured by collecting two consecutive 20-minute urine samples from the bladder cannula. A midpoint arterial blood sample (~200 μL) was also collected in BD Microtainer tubes containing heparin. Urine samples were kept on ice during collection and stored at -20°C until analysis. Blood samples were centrifuged at 6,000 x *g* and 4°C for 5 min. Immediately after centrifugation, plasma was transferred to pre-labelled Eppendorf tubes and stored at -20°C until inulin concentration was measured as described (Miller AJPRFEP 1994, Miller KI 1994). Inulin clearance was calculated as:

$$Inulin\;Clearance=\frac{Urine\;Inulin}{Plasma\;Inulin}\times Urine\;Flow\;Rate$$

Inulin clearance was normalized to total renal mass for each animal.

### Systemic blood pressure

Systemic blood pressure and heart rate were determined in conscious rats with CODA (Kent Scientific Corporation, Torrington, CT), a non-invasive system that measures blood pressure via a tail-cuff. Rats were habituated to the tail-cuff system at least three times on separate days during the week before measurement.

### Cardiac function

Left ventricular function was determined by analyzing pressure-volume loops [25]. After shaving the neck and abdomen, rats were weighed and anesthetized with a cocktail containing ketamine (80–100 mg/kg; Vedco, Inc., St Joseph MO) and xylazine (5–10 mg/kg; Akorn, Inc., Lake Forest, IL). Rats were then placed on a water-circulating heating pad to maintain body temperature and connected to a rodent ventilator (Hugo Saks Elektronik VSM Ventilator Type 698).

Cardiac function measurements were taken with a Millar Pressure-Volume Loop System and a Mikro-Tip Pressure Transducer Catheter (Millar, Inc., Houston, TX). Hemodynamic cardiac parameters were recorded for approximately 30 minutes during steady state, an occlusion test, and volume calibration. The right common carotid artery was exposed, and the catheter was introduced into the vessel through an arteriotomy. After the carotid artery pressure was stable and measured, the catheter was advanced into the left ventricle. Once the proper position of the catheter was confirmed by the shape of the pressure-volume (PV) loops, steady state measurements were recorded.

For the occlusion test, a laparotomy was performed to expose the diaphragm. A small incision was made in the diaphragm to access the inferior vena cava in the thorax. Rubber-tipped forceps were used to occlude the inferior vena cava and reduce venous return to the heart. The forceps were then released to allow blood flow to recover. This occlusion test was repeated 2–4 times in each rat to obtain pressure volume loops at varying preloads.

The raw volume measurement included blood volume in the left ventricle and volume from the surrounding tissues. To selectively obtain the ventricular blood volume, a saline calibration was performed by injecting 50 μL hypertonic saline [15% NaCl (Sigma-Aldrich Co., St Louis, MO)] in 0.9% sodium chloride (Hospira Inc., Lake Forest, IL)] into the inferior vena cava.

### Tissue collection

Animals were perfused with saline (0.5% of body weight; Hospira, Inc., Lake Forest, IL) followed by formalin (Fisherbrand, Pittsburgh, PA) via the left ventricle. The heart (cardiac ventricles without atria and great vessels), left atrium, kidneys, lungs, and epididymal fat pads were harvested and weighed. The heart and kidneys were preserved in 10% neutral buffered formalin (Thermo Fisher Scientific, Hampton, NH) for histological analysis. Lung tissue was dried at 80°C in an oven for 4 days before measuring its dry weight. Pulmonary edema was evaluated based on pulmonary water content calculated as:

$$Water\;Content=\frac{\left(Wet\;Weight-Dry\;Weight\right)}{Wet\;Weight}\times 100$$

### Histology and immunochemistry

Kidneys and hearts fixed in formalin overnight were processed for paraffin embedding and sectioned. Sections of 2 μm thickness were stained with periodic acid-Schiff (PAS) reagent (Electron Microscopy Sciences, Hatfield, PA) or methenamine silver-trichrome (Charles River Laboratories, Inc., Frederick, MD). Sections of 4 μm thickness were stained with hematoxylin and eosin (H&E) reagent (Richard-Allan Scientific, San Diego, CA) or Picrosirius Red (PSR; Polysciences, Warrington, PA), or they were processed for immunohistochemistry. Stained sections were imaged with a Nikon Eclipse E800 Microscope.

For immunohistochemistry, sections of 4 μm thickness were incubated at 60°C for 1 hour, deparaffinized in xylene (Thermo Fisher Scientific, Hampton, NH), and rehydrated stepwise in decreasing concentrations of ethanol (Thermo Fisher Scientific, Hampton, NH). All sections were boiled in antigen retrieval buffer (DAKO Target Retrieval, Dako, Denmark) for 20 min and cooled down to room temperature for 20 min. Sections were blocked for endogenous peroxidase activity with 3% H_2_O_2_ (Sigma-Aldrich, St. Louis, MO) in methanol (Thermo Fisher Scientific, Hampton, NH) for 30 min, washed, and then blocked using an avidin/biotin-blocking kit (DAKO, Denmark). Sections were then incubated with antibody against Kidney Injury Molecule-1 (KIM-1) (Novus Biologicals, Littleton, CO) for 1 hour. The primary antibody signal was detected and amplified with a Tyramide Signal Amplification (TSA) kit according to the manufacturer’s instructions (Perkin Elmar, Waltham, MA). Stained sections were imaged with a Nikon Eclipse E800 Microscope.

### Histological quantification

To quantify renal or cardiac fibrosis, images were taken from five fields of the PSR-stained renal cortex or wall of the left cardiac ventricle of each animal under polarized and bright light (100X magnification). To calculate the percentage of PSR-positive tissue, PSR (under polarized light) and total tissue area (under bright light) were measured with Image-Pro Premier software (Media Cybernetics, Rockville, MD).

To evaluate kidney injury, images of the renal tubules were taken from 10 fields of KIM-1-stained sections for each animal (200X magnification). Renal tubular damage was scored as previously described [26].

To quantify mesangial expansion and glomerulus size, images of glomeruli were taken from six fields of PAS-stained sections from each animal (40X magnification). Increased mesangial matrix was determined by measuring the PAS-positive and nuclei-free area in the mesangium as previously described [27].

To score glomerulosclerosis, microscopic evaluation of sections stained with methenamine silver-trichrome was conducted by a board-certified veterinary pathologist (Charles River Laboratories, Inc., Frederick, MD). The following grades of glomerulosclerosis severity were used: 1 = Minimal, <10% glomeruli affected; 2 = Mild, 10–35% glomeruli affected; 3 = Moderate, 35–65% glomeruli affected; 4 = Marked, 65–90% glomeruli affected; 5 = Severe, >90% glomeruli affected.

To quantify cardiomyocyte size, H&E stained slides of the left ventricle and septum were examined. In each animal, the circumference of 40 and 30 cardiomyocytes were measured from the left ventricle and septum, respectively. Only cardiomyocytes with complete cell boundaries and clear round nuclei were included. Cell circumference was measured by tracing along the cell membrane using Image-Pro Premier software (Media Cybernetics, Rockville, MD)

### Data analysis

All statistical analyses were performed using GraphPad Prism version 5.00 for Windows (San Diego, CA). Data are expressed as mean ± SEM. As appropriate, groups were compared by a t-test, one-way ANOVA followed by a post hoc Dunnett’s test, or two-way ANOVA followed by a post hoc Bonferroni test. A value of *P* < 0.05 was considered statistically significant.

## Results

### Characterization of ZSF1 rats

Before FG-2216 treatment, baseline parameters were determined for lean ZSF1 rats that underwent a sham procedure (Ln-ZSF1) and obese ZSF1 rats that underwent unilateral nephrectomy (Nx) to accelerate the development of nephropathy (Nx-Ob-ZSF1). Three weeks after nephrectomy, Nx-Ob-ZSF1 rats exhibited significantly higher body weight, serum blood urea nitrogen (BUN), serum creatinine, serum glucose, and serum cholesterol than Ln-ZSF1 rats. Nx-Ob-ZSF1 rats also showed elevated proteinuria, glycosuria and urine sodium excretion, as well as increased diastolic and mean blood pressure. Their hemoglobin level was not different from that of Ln-ZSF1 rats (Table 1).

**Table 1:** Baseline characteristics of Ln-ZSF1 and Ob-ZSF1 rats.

Parameter	Ln-ZSF1	Nx-Ob-ZSF1
Body weight (g)	335 ± 7	506 ± 4*
Hemoglobin (g/dL)	15.2 ± 0.46	15.7 ± 0.1
Serum BUN (mg/dL)	20 ± 0.6	32 ± 0.7*
Serum creatinine (mg/dL)	0.44 ± 0.02	0.58 ± 0.01*
Serum cholesterol (mg/dL)	70 ± 2	171 ± 5*
Serum glucose (mg/dL)	173 ± 8	484 ± 23*
Proteinuria (mg/24 hours)	1.53 ± 0.10	3.52 ± 0.31*
Glycosuria (mg/24 hours)	1.75 ± 0.12	82 ± 41*
Urine sodium (mEq/24hrs)	0.53 ± 0.05	1.30 ± 0.09*
SBP (mmHg)	169 ± 3	181 ± 4
DBP (mmHg)	124 ± 4	141 ± 4*
MBP (mmHg)	139 ± 4	155 ± 4*

BUN, blood urea nitrogen; DBP, diastolic blood pressure; MBP, mean blood pressure; SBP, systolic blood pressure.

Mean ± SEM; n = 7–9 animals/Ln-ZSF1 group & n = 22–24 animals/Nx-Ob-ZSF1 group

*P < 0.05 vs. Ln-ZSF1 rats (t-test).

### FG-2216 prevented the reduction in blood hemoglobin in Nx-Ob-ZSF1 rats

**Fig 2:**
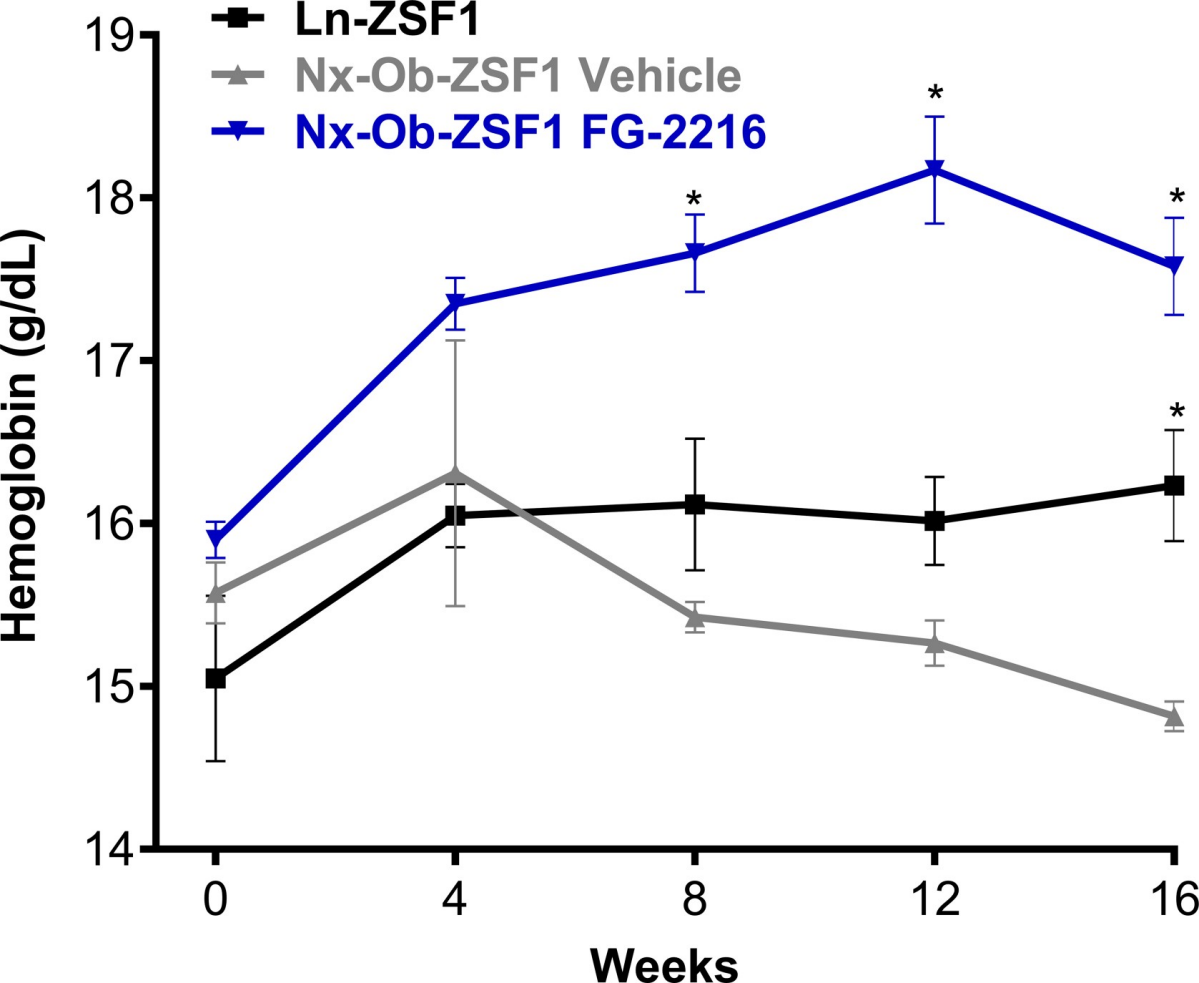
FG-2216 corrected hemoglobin levels in Nx-Ob-ZSF1 rats. Hemoglobin levels were determined from blood collected every four weeks during treatment. Values represent mean ± SEM (n = 6–12). *P < 0.05 *vs*. Nx-Ob-ZSF1 Vehicle (Bonferroni test).

After 16 weeks of treatment, Nx-Ob-ZSF1 Vehicle rats had significantly lower blood hemoglobin levels than Ln-ZSF1 rats. FG-2216 significantly increased hemoglobin levels in Nx-Ob-ZSF1 rats starting after 8 weeks of treatment (Fig 2). FG-2216 also significantly increased hematocrit, mean corpuscular volume and mean corpuscular hemoglobin without affecting red blood cell count or mean corpuscular hemoglobin concentration in Nx-Ob-ZSF1 rats (S1 Table).

### FG-2216 enhanced kidney function in Nx-Ob-ZSF1 rats

**Fig 3:**
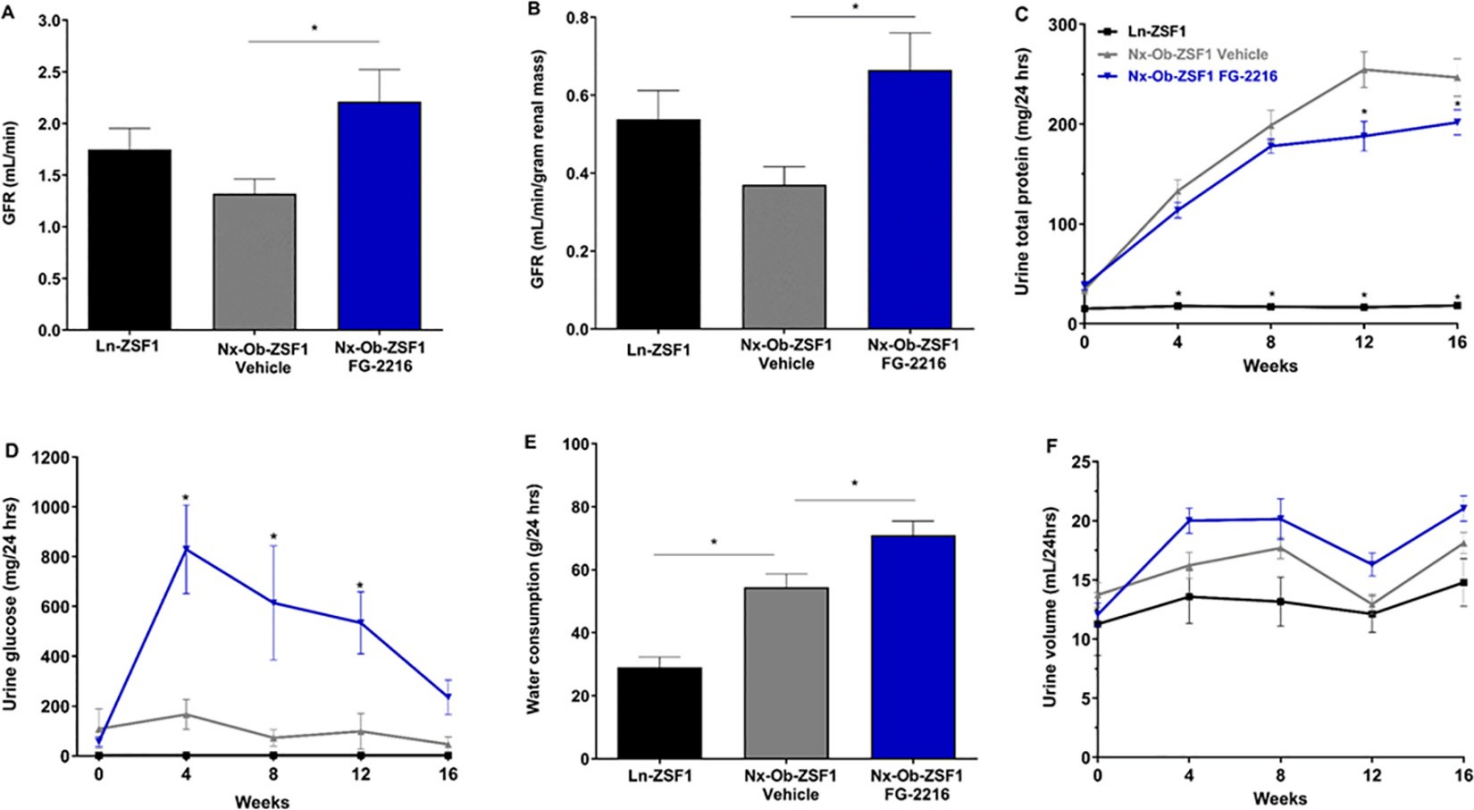
FG-2216 improved kidney function in Nx-Ob-ZSF1 rats. Glomerular filtration rate (GFR) was determined by inulin clearance assay after 18 weeks of treatment (*A*). GFR was normalized to total renal mass (*B*). Urine was collected for 24 hours every four weeks during the treatment period to measure proteinuria (*C)*, glycosuria (*D*) and volume (*F*). After 17 weeks of treatment, water consumption was measured over 48 hours encompassing a dosing and a non-dosing day (*E*). Values represent mean ± SEM (n = 7–12). *P < 0.05 *vs*. Nx-Ob-ZSF1 Vehicle (Dunnett’s test for A, B, and E; Bonferroni test for C, D and F).

After 18 weeks of treatment, Nx-Ob-ZSF1 FG-2216 rats showed a significantly improved GFR as measured by inulin clearance (Fig 3*A* and 3*B*) and significantly decreased proteinuria (Fig 3*C*) compared to Nx-Ob-ZSF1 Vehicle rats. Nx-Ob-ZSF1 rats treated with FG-2216 also had significantly higher urine glucose excretion than vehicle-treated Nx-Ob-ZSF1 rats, although the magnitude of the increase in glycosuria diminished over time (Fig 3*D*). Nx-Ob-ZSF1 Vehicle rats drank more water than Ln-ZSF1 rats and water consumption was further increased in Nx-Ob-ZSF1 FG-2216 rats (Fig 3*E*). Urine volume tended to increase in Nx-Ob-ZSF1 Vehicle compared to Ln-ZSF1 rats and to further increase in Nx-Ob-ZSF1 FG-2216 rats but these difference did not reach statistical significance (Fig 3*F*). FG-2216 did not affect elevated levels of serum creatinine and BUN in Nx-Ob-ZSF1 rats (S1*A* and S1*B* Fig). FG-2216 also did not significantly affect urine creatinine levels compared to vehicle in Nx-Ob-ZSF1 rats over the course of the study except at the 12-week time point (S1*C* Fig). Urine sodium excretion was higher in the Nx-Ob-ZSF1 Vehicle than in the Ln-ZSF1 rats early in the study but the difference between the two groups diminished over the course of the study (S1*D* Fig). FG-2216 increased urine sodium excretion in Nx-Ob-ZSF1 rats compared to vehicle in the later part of the study (S1*D* Fig).

### FG-2216 reduced kidney injury in Nx-Ob-ZSF1 rats

**Fig 4:**
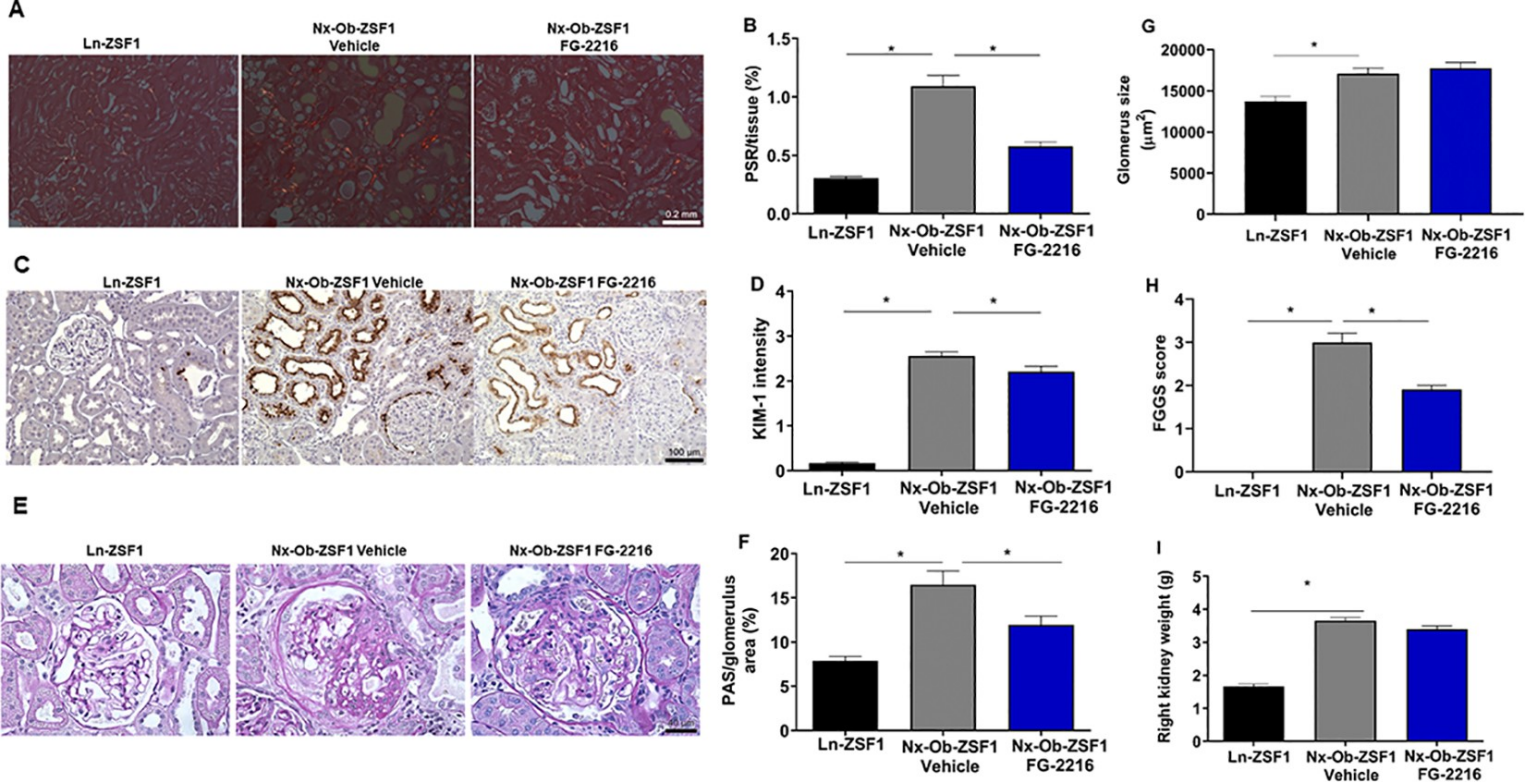
FG-2216 improved kidney morphology in Nx-Ob-ZSF1 rats. Kidneys were harvested after 18 weeks of treatment and sectioned for histology. Picrosirius red (PSR) staining of fibrillar collagen showed tubular interstitial fibrosis (*A*). FG-2216 reduced PSR staining (*B*). Immunohistochemistry for Kidney Injury Molecule-1 (KIM-1) indicated tubular damage (*C*). FG-2216 decreased KIM-1 signal intensity (*D*). Periodic acid-Schiff (PAS) staining showed mesangial expansion in the glomerulus (*E*). FG-2216 reduced fractional mesangial expansion (*F*) without affecting glomerulus tuft area (*G*). Glomerulosclerosis quantified on sections stained with methenamine silver-trichrome showed less focal global glomerulosclerosis (FGGS) in Nx-Ob-ZSF1 FG-2216 animals than in Nx-Ob-ZSF1 Vehicle animals (*H*). The right kidney increased in mass in the Nx-Ob-ZSF1 animals to compensate for nephrectomy of the left kidney (*I*). Values represent mean ± SEM (n = 8–12). *P < 0.05 *vs*. Nx-Ob-ZSF1 Vehicle (Dunnett’s test).

PSR staining showed more fibrillar collagen in the tubulointerstitial space of Nx-Ob-ZSF1 Vehicle rats than in Ln-ZSF1 rats, which was reduced by FG-2216 treatment (Fig 4*A* and 4*B*). Tubular damage in renal tissue was assessed with immunohistochemistry by quantifying the levels of KIM-1, a specific and sensitive marker of kidney injury [26]. Compared to Ln-ZSF1 rats, Nx-Ob-ZSF1 Vehicle rats showed significantly elevated levels of KIM-1 in the kidney, which were significantly reduced by FG-2216 treatment (Fig 4*C* and 4*D*). Histopathology of renal glomeruli was evaluated with PAS and methenamine silver-trichrome staining. PAS staining showed that Nx-Ob-ZSF1 Vehicle rats displayed increased glomerulus size and fractional mesangial area within the glomeruli compared to Ln-ZSF1 rats. FG-2216 treatment reduced mesangial expansion without affecting glomerulus size (Fig 4*E*–4*G*). Methenamine silver-trichrome staining showed that no glomerulosclerosis was present in Ln-ZSF1 animals but that all animals in both Nx-Ob-ZSF1 groups had focal segmental glomerulosclerosis (FSGS) and focal global glomerulosclerosis (FGGS). There were few glomeruli with FSGS in the two Nx-Ob-ZSF1 groups. The predominant change was FGGS which was more prevalent in glomeruli at the corticomedullary junction than in the outer cortex. The severity of FGGS was significantly less in the Nx-Ob-ZSF1 FG-2216 group than in the Nx-Ob-ZSF1 Vehicle group (Fig 4*H*). As expected, nephrectomy of the left kidneys resulted in significantly larger right kidneys in both Nx-Ob-ZSF1 Vehicle and Nx-Ob-ZSF1 FG-2216 rats than in non-nephrectomized Ln-ZSF1 rats (Fig 4*I*). These results show that FG-2216 improved kidney histopathology by decreasing tubular interstitial fibrosis, tubular damage, mesangial expansion and glomerulosclerosis in Nx-Ob-ZSF1 rats.

### FG-2216 did not affect glucose tolerance in Nx-Ob-ZSF1 rats

**Fig 5:**
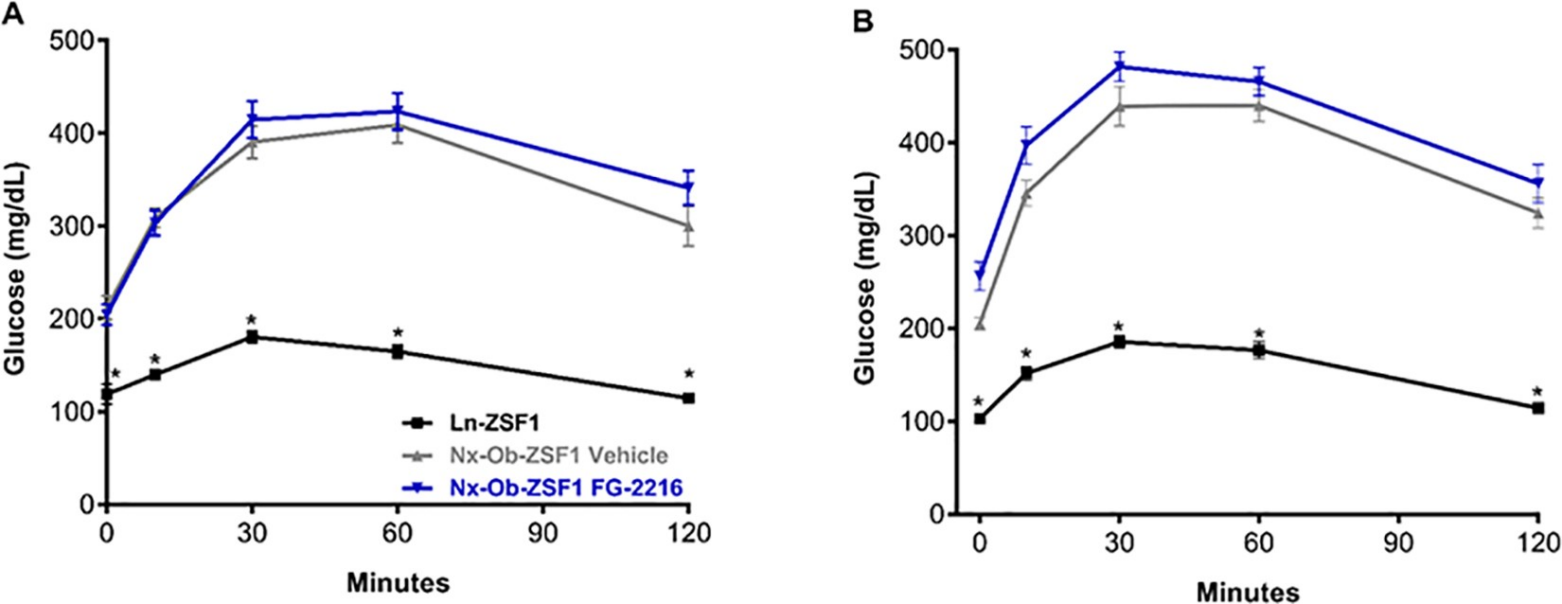
FG-2216 did not affect glucose tolerance in Nx-Ob-ZSF1 rats. Blood glucose levels during oral glucose tolerance tests after 6 weeks (*A*) and 14 weeks (*B*) of treatment. Values represent mean ± SEM (n = 8–12). *P < 0.05 *vs*. Nx-Ob-ZSF1 Vehicle (Bonferroni test).

Oral glucose tolerance tests (OGTTs) were conducted after 6, 10, and 14 weeks of treatment to examine the effect of FG-2216 on glucose handling by Nx-Ob-ZSF1 rats. Before glucose loading, Nx-Ob-ZSF1 Vehicle and Nx-Ob-ZSF1 FG-2216 rats had similar fasting glycemia that was higher than that of Ln-ZSF1 rats. During the OGTTs, Nx-Ob-ZSF1 Vehicle and Nx-Ob-ZSF1 FG-2216 rats showed similar blood glucose excursion after 6 (Fig 5*A*), 10 (S2 Fig), and 14 weeks of treatment (Fig 5*B*). Blood glucose peaked at 30–60 minutes into the OGTTs in all groups, and glucose levels were not significantly different between Nx-Ob-ZSF1 FG-2216 and Nx-Ob-ZSF1 Vehicle rats at any time point.

### FG-2216 reduced obesity in Nx-Ob-ZSF1 rats

**Fig 6:**
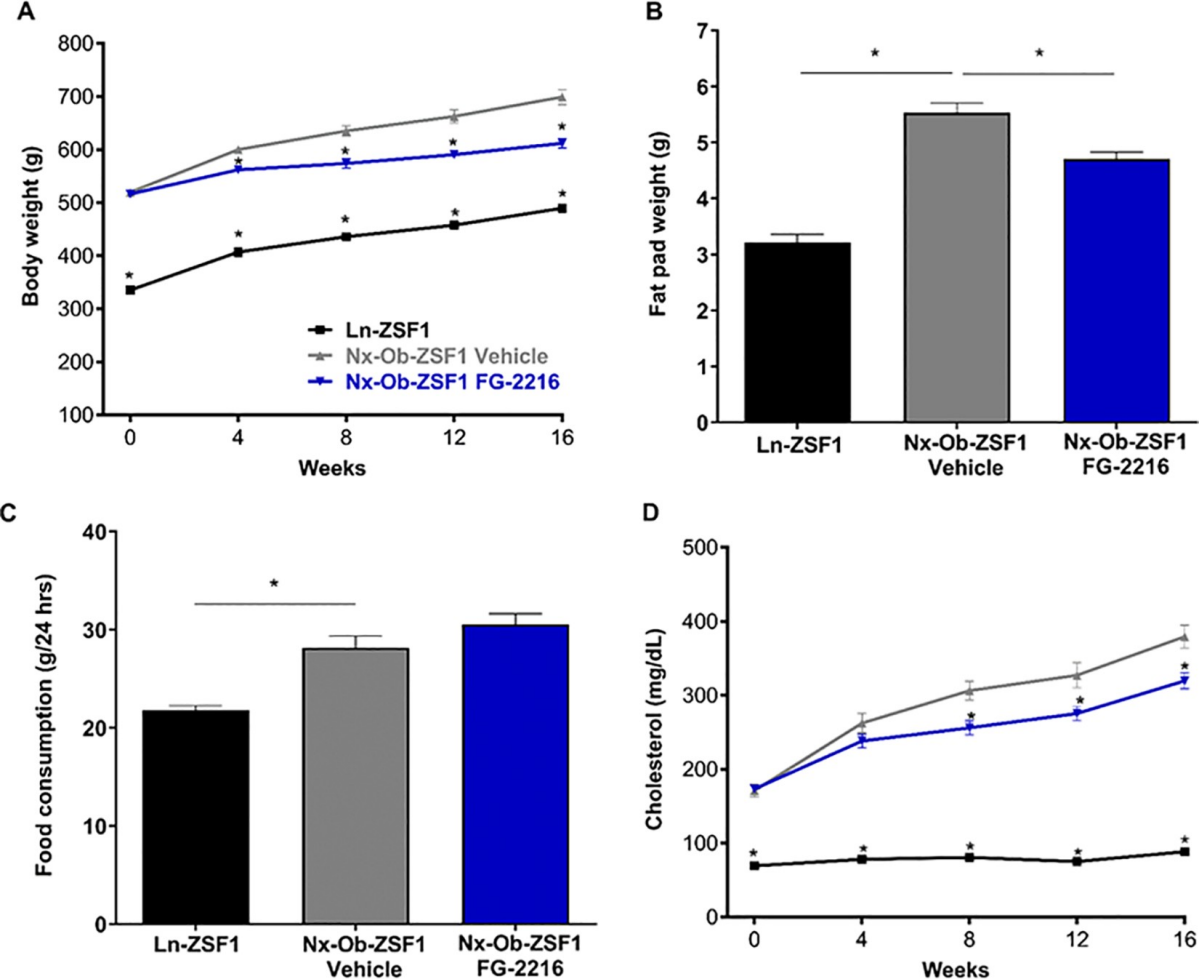
FG-2216 reduced obesity in Nx-Ob-ZSF1 rats. Rats were weighed at regular intervals during the treatment period (*A*). Fat pads were harvested after 18 weeks of treatment and weighed (*B*). After 17 weeks of treatment, food consumption was measured over 48 hours encompassing a dosing and a non-dosing day (*C*). Blood was collected every four weeks during the treatment period to determine serum cholesterol (*D*). Values represent mean ± SEM (n = 8–12). *P < 0.05 *vs*. Nx-Ob-ZSF1 Vehicle (Bonferroni test for A and D; Dunnett’s test for B and C).

All groups gained weight over the study period. As expected, Nx-Ob-ZSF1 Vehicle rats had larger body weight and heavier fat pad than Ln-ZSF1 rats (Fig 6*A* and 6*B*). After 18 weeks of treatment, Nx-Ob-ZSF1 FG-2216 rats had significantly lower body weight (Fig 6*A*) and epididymal fat pad weight than Nx-Ob-ZSF1 Vehicle rats (Fig 6*B*). Nx-Ob-ZSF1 Vehicle rats were hyperphagic compared to Ln-ZSF1 rats (Fig 6*C*). Nx-Ob-ZSF1 Vehicle and Nx-Ob-ZSF1 FG-2216 rats ate similar amounts of food (Fig 6*C*), indicating that FG-2216 reduced obesity independently of food consumption.

Nx-Ob-ZSF1 Vehicle rats had increased serum cholesterol levels compared with Ln-ZSF1 rats, which was reduced by FG-2216 treatment (Fig 6*D*). No difference in serum triglyceride levels were observed between Nx-Ob-ZSF1 FG-2216 and Nx-Ob-ZSF1 Vehicle rats (S3 Fig).

### FG-2216 reduced hypertension and cardiac hypertrophy in Nx-Ob-ZSF1 rats

**Fig 7:**
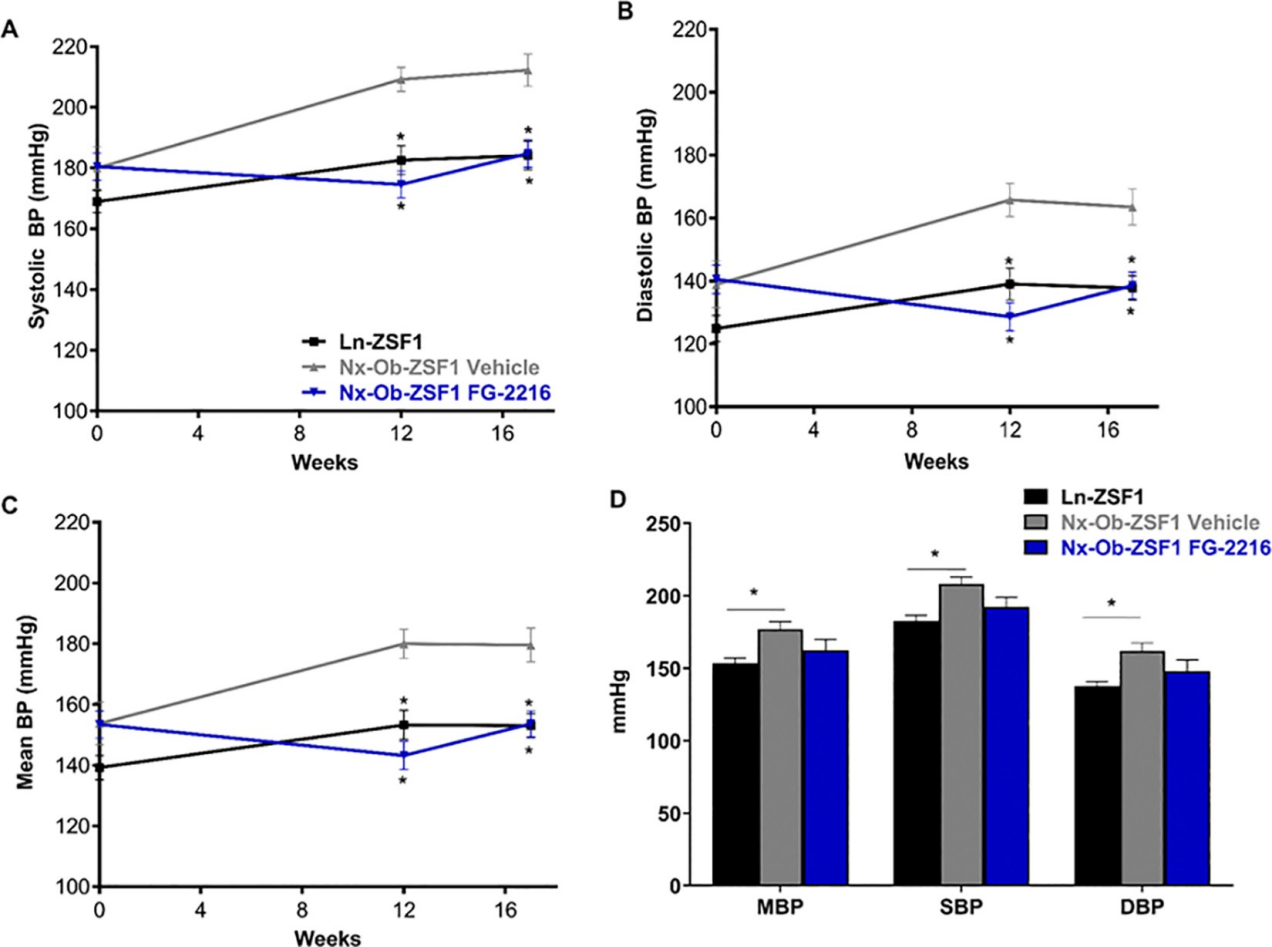
FG-2216 prevented hypertension in Nx-Ob-ZSF1 rats. Systolic (*A*) and diastolic (*B*) blood pressures (BP) were measured and mean BP (*C*) was calculated using a tail-cuff at baseline and three hours after dose administration at 12 and 17 weeks of treatment. Systolic (SBP) and diastolic (DBP) blood pressures were also measured 24 hours following dosing after 17 weeks of treatment and mean BP (MBP) was calculated (*D*). Values represent mean ± SEM (n = 8–12). *P < 0.05 *vs*. Nx-Ob-ZSF1 Vehicle (Bonferroni test).

At 12 and 17 weeks of treatment, FG-2216 corrected the elevated systolic and diastolic blood pressure observed in Nx-Ob-ZSF1 rats 3 hours after dose administration (Fig 7*A* and 7*B*), thereby significantly reducing mean blood pressure in these animals (Fig 7*C*). This reduction was maintained 24 hours after administration but did not reach statistical significance at that time point (Fig 7*D*). All groups exhibited similar heart rates at the times of blood pressure measurement (S4 Fig).

**Fig 8:**
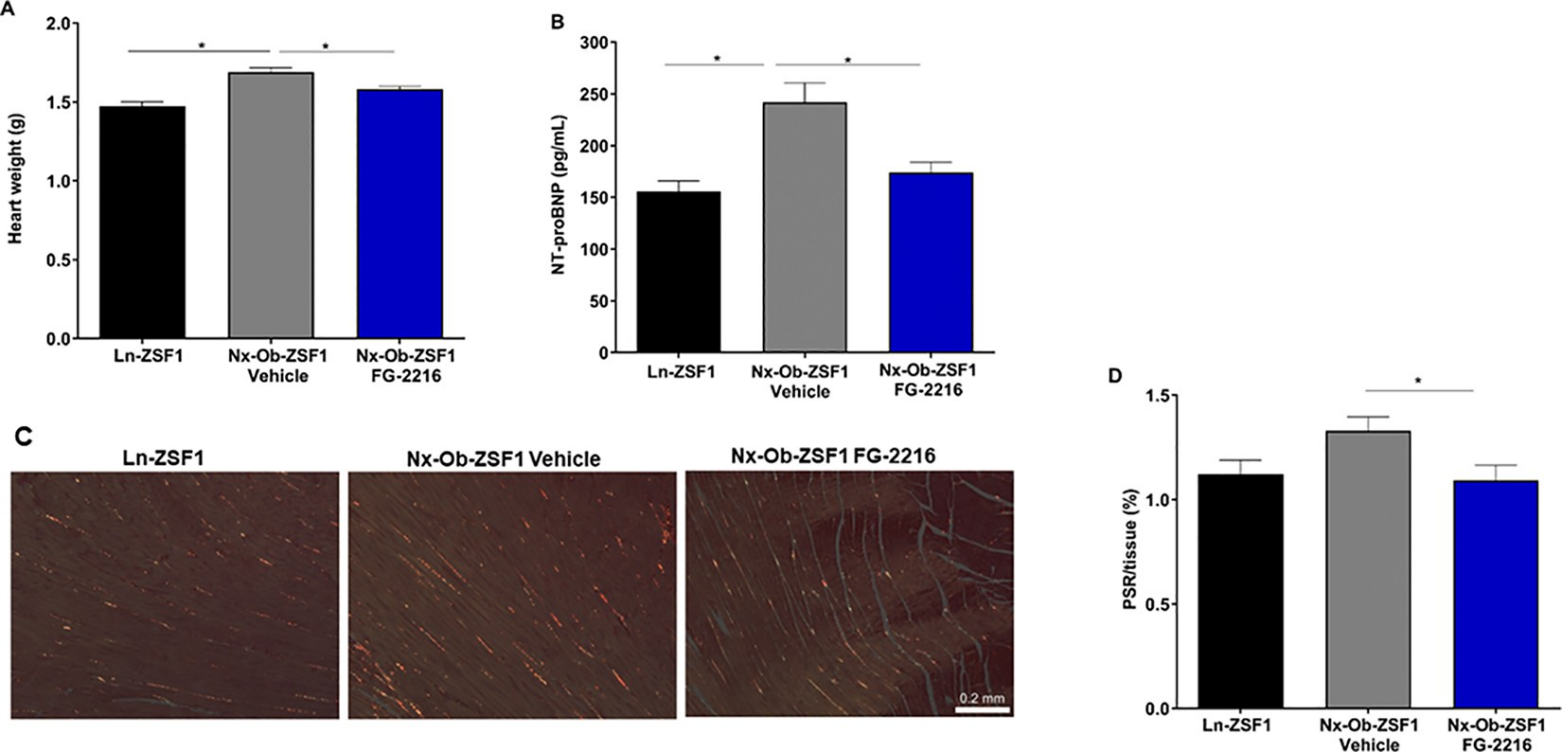
FG-2216 reduced cardiac hypertrophy in Nx-Ob-ZSF1 rats. Hearts were harvested and weighed after 18 weeks of treatment. FG-2216 normalized heart weight in Nx-Ob-ZSF1 rats (*A*). Blood was collected from rats to measure plasma levels of NT-proBNP after 16 weeks of treatment. FG-2216 normalized NT-proBNP concentration in Nx-Ob-ZSF1 rats (*B*). Cardiac histology with picrosirius red (PSR) staining for fibrillar collagen showed left ventricular fibrosis (*C*). FG-2216 decreased PSR staining in Nx-Ob-ZSF1 rats (*D*). Values represent mean ± SEM (n = 8–12). *P < 0.05 *vs*. Nx-Ob-ZSF1 Vehicle (Dunnett’s test).

Nx-Ob-ZSF1 Vehicle rats had significantly increased heart weight compared to Ln-ZSF1 rats after 18 weeks of treatment. FG-2216 significantly reduced heart weight in Nx-Ob-ZSF1 rats (Fig 8*A*). Pulmonary water content, an indicator of pulmonary edema and heart failure, was not different between any of the groups (S5 Fig). Nx-Ob-ZSF1 Vehicle rats had significantly higher plasma NT-proBNP (a marker of cardiac hypertrophy) than Ln-ZSF1 rats, which was significantly reduced by FG-2216 treatment (Fig 8*B*). Nx-Ob-ZSF1 Vehicle rats had more cardiac interstitial fibrosis measured by PSR staining in the left ventricle than Ln-ZSF1 rats, and Nx-Ob-ZSF1 FG-2216 rats had significantly less fibrosis than Nx-Ob-ZSF1 Vehicle rats (Fig 8*C* and 8*D*). No difference in cardiomyocyte size was observed among groups in the left ventricle wall or the septum (S6*A* and S6*B* Fig).

**Fig 9:**
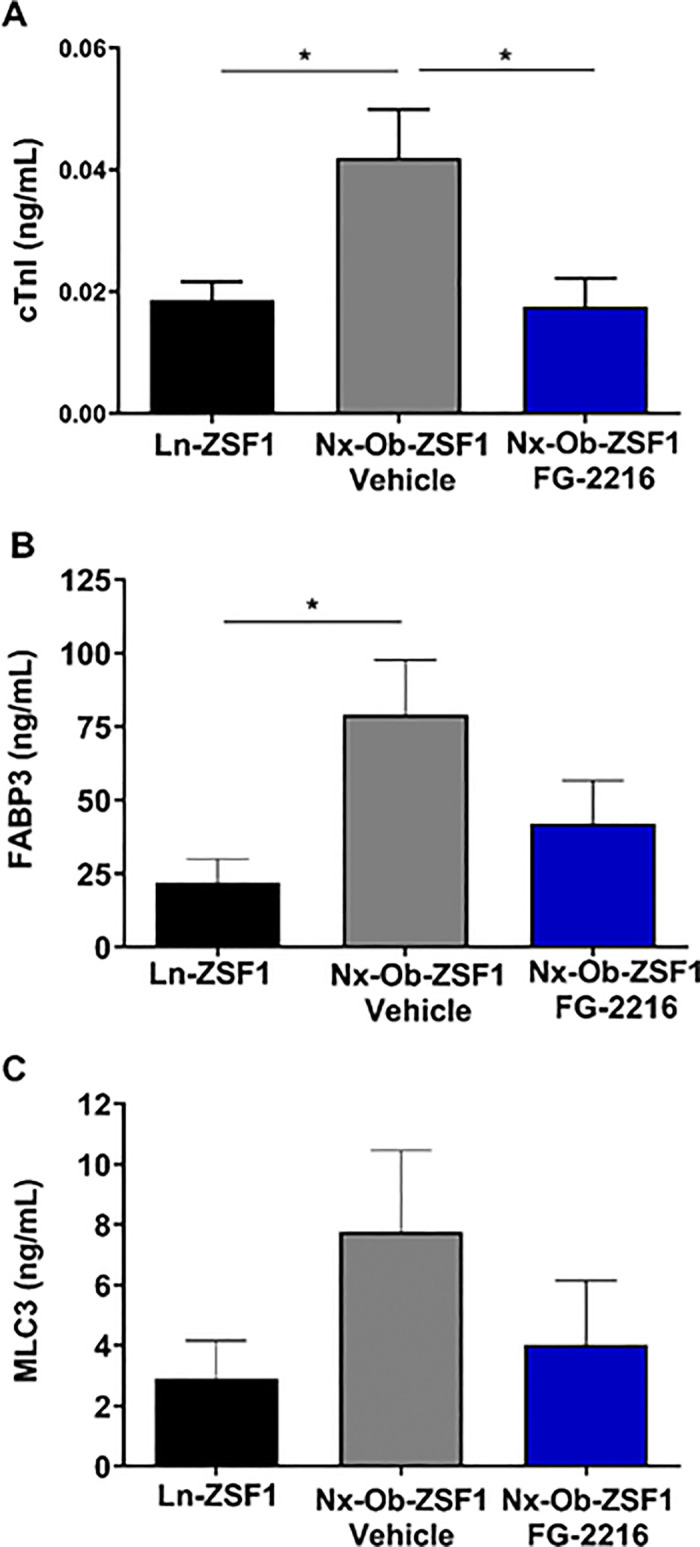
FG-2216 decreased plasma markers of cardiac injury in Nx-Ob-ZSF1 rats. Blood was collected after 16 weeks of treatment to measure plasma concentrations of cardiac troponin I (cTnI) (*A*), fatty acid binding protein 3 (FABP3) (*B*), and myosin light chain 3 (MLC3) (*C*). Values represent mean ± SEM (n = 8–12). *P < 0.05 *vs*. Nx-Ob-ZSF1 Vehicle (Dunnett’s test).

After 16 weeks of treatment, Nx-Ob-ZSF1 Vehicle rats had increased plasma levels of cardiac troponin I (cTnI) compared with Ln-ZSF1 rats, and FG-2216 significantly decreased circulating cTnI concentration in Nx-Ob-ZSF1 rats (Fig 9*A*). The plasma levels of two other markers of cardiac injury, fatty acid binding protein 3 (FABP3; Fig 9*B*) and myosin light chain 3 (MLC3; Fig 9*C*), were also lower in Nx-Ob-ZSF1 FG-2216 rats than Nx-Ob-ZSF1 Vehicle rats, but the differences were not statistically significant.

### FG-2216 improved cardiac function in Nx-Ob-ZSF1 rats

Left ventricular function was assessed in Nx-Ob-ZSF1 rats in a separate (cardiac function) study. Because Ln-ZSF1 rats are hypertensive, and hypertension affects the development of heart failure, we used normotensive Wistar-Kyoto (WKY) rats instead of Ln-ZSF1 rats for reference, as suggested in previous work [23, 28, 29]. Nx-Ob-ZSF1 Vehicle rats exhibited increased end-diastolic pressure (EDP) and isovolumetric relaxation time (IVRT) compared to WKY control rats (Table 2). End-diastolic volume (EDV) was also elevated in Nx-Ob-ZSF1 Vehicle rats compared to WKY rats but the difference was not statistically significant. FG-2216 significantly reduced EDP, IVRT and EDV in Nx-Ob-ZSF1 rats. Nx-Ob-ZSF1 Vehicle rats also exhibited increased end-systolic pressure and volume, and decreased ejection fraction compared to WKY control rats (Table 2). FG-2216 significantly improved these three parameters in Nx-Ob-ZSF1 rats. FG-2216 also decreased the elevated arterial elastance (Ea) and total peripheral resistance observed in Nx-Ob-ZSF1 Vehicle rats, but this effect was not statistically significant. No differences in stroke volume, heart rate, cardiac output, dP/dt minimum, dP/dt maximum, end-diastolic pressure-volume relationship or end-systolic pressure-volume relationship were observed among groups. Systemic blood pressure in the carotid artery was also measured with the intravascular Millar catheter at the time of the cardiac function measurement, 2–4 days after administering the last dose of the compound. FG-2216 significantly decreased systolic and diastolic blood pressures in Nx-Ob-ZSF1 rats (Table 2).

**Table 2:** FG-2216 improved cardiac function in Nx-Ob-ZSF1 rats.

	WKY	Nx-Ob-ZSF1 Vehicle	Nx-Ob-ZSF1 FG-2216
ESP (mmHg)	119 ± 3*	161 ± 3	140 ± 4*
EDP (mmHg)	5 ± 0.4*	13 ± 1.0	7 ± 0.7*
ESV (μL)	155 ± 23*	296 ± 28	192 ± 29*
EDV (μL)	392 ± 17	473 ± 29	360 ± 34*
EF (%)	79 ± 4.3*	61 ± 4.1	75 ± 3.5*
SV (μL)	300 ± 13	293 ± 17	289 ± 14
HR (bpm)	160 ± 3	159 ± 3	163 ± 4
CO (μL/min)	47956 ± 2317	46680 ± 2729	47183 ± 2681
IVRT (msec)	13 ± 0.42*	17 ± 0.68	15 ± 0.26*
dP/dt min (mmHg/sec)	-7232 ± 267	-8606 ± 682	-7983 ± 625
dP/dt max (mmHg/sec)	9172 ± 307	9303 ± 228	9206 ± 293
EDPVR (mmHg/μL)	0.018 ± 0.001	0.016 ± 0.002	0.019 ± 0.002
ESPVR (mmHg/μL)	0.48 ± 0.07	0.47 ± 0.06	0.47± 0.07
Ea (mmHg/μL)	0.41 ± 0.02*	0.58 ± 0.04	0.50 ± 0.03
TPR (mmHg.min/mL)	2.1 ± 0.12*	2.9 ± 0.23	2.5 ± 0.14
SBP (mmHg)	135 ± 4*	177 ± 3	154 ± 3*
DBP (mmHg)	80 ± 3*	104 ± 2	93 ± 4*

CO, cardiac output; DBP, diastolic blood pressure; EDP, end-diastolic pressure; EDPVR, end-diastolic pressure-volume relationship; EDV, end-diastolic volume; Ea, arterial elastance; EF, ejection fraction; ESP, end-systolic pressure; ESPVR, end-systolic pressure-volume relationship; ESV, end-systolic volume; HR, heart rate; IVRT, isovolumetric relaxation time; SBP, systolic blood pressure; SV, stroke volume; TPR, total peripheral resistance; WKY, Wistar-Kyoto.

Mean ± SEM; n = 9 animals/WKY group; n = 14–15 animals/Nx-Ob-ZSF1 Vehicle group; n = 11–12 animals/Nx-Ob-ZSF1 FG-2216 group

*P <0 .05 vs. Nx-Ob-ZSF1 Vehicle (Dunnett’s test).

In this follow-up cardiac study, we also confirmed several of the findings observed in the initial (renal function) study. Nx-Ob-ZSF1 Vehicle rats displayed decreased levels of hemoglobin and increased body weight, epididymal fat pad weight, and serum cholesterol compared to WKY rats. FG-2216 improved all these parameters in Nx-Ob-ZSF1 rats. Compared to WKY rats, Nx-Ob-ZSF1 Vehicle rats also exhibited increased heart weight and plasma levels of the cardiac hypertrophy marker NT pro-BNP, which were both reduced by treatment with FG-2216 in Nx-Ob-ZSF1 rats. There was no change in pulmonary water content between groups (S2 Table).

## Discussion

HIF-PHIs are currently being developed as novel therapeutics for treating anemia, a common complication of kidney disease characterized by decreased levels of circulating hemoglobin [11, 14]. Activation of the HIF pathway by HIF-PHIs corrects low hemoglobin levels in several animal models, including mice, rats, and non-human primates [16–19]. It also improves anemia in patients with CKD [7, 15]. Consistent with these findings, we showed that the HIF-PHI FG-2216 prevented the decrease in blood hemoglobin concentration observed in Nx-Ob-ZSF1 rats.

Patients with CKD often suffer from other comorbidities, including hypertension, heart disease, diabetes, and obesity [30, 31]. Ob-ZSF1 rats exhibit many of the clinical manifestations seen in patients with diabetic nephropathy, including obesity, dyslipidemia, hypertension, proteinuria, renal fibrosis, and cardiomyopathy [24]. Because HIF activation has been shown to provide cardiac and renal protection in animal models [32–35], we hypothesized that the HIF-PHI FG-2216 may also improve comorbidities associated with kidney disease.

Long-term (18 weeks) administration of FG-2216 significantly improved kidney function, including GFR and proteinuria, as well as renal histopathological changes including fractional mesangial expansion within glomeruli, glomerulosclerosis, tubulointerstitial fibrosis, and tubular injury in Nx-Ob-ZSF1 rats. Glomeruli were larger and exhibited an increased fractional mesangial area as well as glomerulosclerosis in Nx-Ob-ZSF1 Vehicle rats compared to Ln-ZSF1 rats. The disproportionate increase in mesangium within hypertrophic glomeruli may have been responsible for the reduced GFR observed in Nx-Ob-ZSF1 Vehicle rats by decreasing capillary filtering surface area [36–38]. Treatment with FG-2216 decreased fractional mesangial areas in Nx-Ob-ZSF1 rats, showing that the compound may have increased GFR by improving filtration surface area. Glomerular size was unaffected by FG-2216, indicating that the increase in GFR observed in Nx-Ob-ZSF1 FG-2216 rats compared to Nx-Ob-ZSF1 Vehicle rats was not due to glomerular enlargement. Glomerulosclerosis was present in Nx-Ob-ZSF1 Vehicle as observed by others in this model [23, 24, 39, 40] and may have been responsible for the marked proteinuria exhibited by these animals since both proteinuria and glomerulosclerosis were reduced in the Nx-Ob-ZSF1 FG-2216 animals [41].

Hyperglycemia may directly influence renal function in diabetic patients and it has been suggested that hyperglycemia causes hyperfiltration in 32-week old ZSF1 rats [36, 39]. FG-2216 did not affect fasting circulating glucose levels or glucose utilization during glucose tolerance tests performed three times over the course of the study. Therefore, it is unlikely that the improvement in GFR observed after treatment with FG-2216 was the direct results of changes in glycemia. Hypertension is another strong independent risk factor for end-stage renal disease and blood pressure control is recommended to prevent kidney failure [42]. The present study confirmed than both lean and obese ZSF1 rats have elevated blood pressure, and obese rats more so than lean rats [28]. Because obese ZSF1 rats have higher blood pressure than lean ZSF1, nephropathy in obese ZSF1 rats may be in part driven by hypertension. Treatment with antihypertensive drugs such as the angiotensin-converting enzyme captopril and the angiotensin receptor blocker cardestan have shown that reduction in blood pressure is accompanied by improvement in renal dysfunction in obese ZSF1 rats [43, 44]. Thus, the antihypertensive effects of FG-2216 observed in this study may also have contributed to improvement in kidney function and renal histopathology in Nx-Ob-ZSF1 FG-2216 rats. Recently, the HIF-PHIs roxadustat and molidustat have been shown to acutely (4 to 6 hours after dosing the animals) reduce renal vascular resistance, and increase renal blood flood flow and GFR in normal rats [45]. These renal hemodynamic changes were mediated in part by nitric oxide generation. Similar mechanisms may have taken place in our study, however we measured GFR three to five days after administration of the last dose of FG-2216, so it is unlikely that acute effects of the compound were involved in the improvement in GFR that we observed.

FG-2216 is a HIF-PHI that increases HIF-α levels in the heart, kidney and liver [20, 21]. In the present study, FG-2216 stimulated hematopoiesis which indicated increased HIF levels and induction of the HIF-target gene EPO in the kidney. The improvement in GFR by FG-2216 agrees with studies showing that HIF activation enhanced renal function in models of acute and chronic kidney injury [34, 35]. HIF activation with cobalt chloride improved GFR in a rat remnant kidney model [46], as well as GFR and kidney fibrosis in diabetic rats [47, 48]. The ability of HIF activation to normalize glomerular filtration may be due to prevention of diabetes-induced changes in oxygen metabolism and reduction of oxidative stress in the kidney [47]. FG-2216 improved proteinuria in Nx-Ob-ZSF1 rats, consistent with studies showing that HIF activation by dimethyloxalylglycine (DMOG) or cobalt chloride reduced proteinuria and kidney fibrosis in a rat remnant kidney model [49, 50] and in type 2 diabetic rats [47, 51]. The HIF-PHI enarodustat also decreased proteinuria in BTBR *ob/ob* mice without affecting glomerular filtration in this model of type 2 diabetes [52].

A major pathological characteristic of renal disease is kidney fibrosis [53, 54]. Nx-Ob-ZSF1 rats had increased kidney tubulointerstitial fibrosis compared with Ln-ZSF1 rats as reported in previous studies [24]. FG-2216 decreased fibrosis in Nx-Ob-ZSF1 rats, consistent with other rodent models showing that activating the HIF pathway reduced renal fibrosis [50, 55, 56]. HIF may regulate a similar effect in humans, because HIF-1α expression inversely correlated with renal interstitial fibrosis in CKD patients [57]. HIF-PHIs may affect kidney fibrosis through several mechanisms, including gene transcription, signaling pathways, the epithelial-mesenchymal transition, and epigenetics [58]. Specifically, HIF-PHIs regulate genes associated with fibrosis, such as tissue inhibitor of metalloproteinase 1, connective tissue growth factor, and plasminogen activator inhibitor 1 [59–61]. HIF-1α activation also upregulated miR-29c, which reduced renal interstitial fibrosis in a rat remnant kidney model [62]. Inhibition of fibrosis by PHIs may also occur independently of the HIF pathway [63].

Nearly all types of CKD are characterized by damage to renal tubule [64]. Nx-Ob-ZSF1 rats displayed increased tubular damage compared to Ln-ZSF1 rats. FG-2216 treatment reduced tubular injury, consistent with reports showing that inhibiting HIF degradation with cobalt chloride or HIF-PHIs reduced tubular necrosis in rat models of kidney injury [50, 65–68]. The mechanisms responsible for HIF-PHI protection against tubular injury are unknown. However, several hypotheses have been suggested, including a reduced number of apoptotic cells in a nephritis model [56] or changes in oxygen metabolism, mitochondrial leak respiration, and kidney hypoxia in a diabetes model [47]. Schley et al. also showed that HIF accumulation reduced proximal tubular injury in mice by enhancing glycolytic enzymes and lactate metabolism [69]. While these studies do not converge on a precise mechanism by which the HIF pathway prevents tubular damage, they support that HIF regulation is a promising approach for renoprotection.

Increase in the HIF target gene vascular endothelial growth factor (VEGF) has been shown to be associated with the obese ZSF1 cardio-renal phenotype [24]. In aged obese ZSF1 rats with advanced renal disease, chronic treatment with 2-methoxyestradiol and its structural analog 2-ethoxyestradiol downregulated renal VEGF expression and provided renal protection [70]. Treatment with growth differentiation factor 15 decreased body weight, food intake, blood glucose, and triglycerides and improved exercise capacity in obese ZSF1 males while decreasing VEGF [71]. VEGF is a marker of vascular injury and inflammation, and as such may not be directly responsible for ZSF1 comorbidities but may help monitor disease progression and correction by therapeutic agents [24]. Activation of the HIF pathway by HIF-PHIs induces considerably lower VEFG mRNA tissue expression or increase in VEGF plasma levels compared to other HIF target genes such as EPO [17, 72]. Consistent with these observations, no effect of HIF-PHIs has been observed on VEGF-dependent tumor models [73]. Some HIF target genes like VEGF are sensitive to hydroxylation of HIF by factor inhibiting HIF (FIH) and may not be optimally induced unless both HIF-PH enzymes and FIH are inhibited [74, 75]. Thus, increases in VEGF expression by activation of the HIF pathway in response to treatment with FG-2216 were probably modest in our study and unlikely to have affected the results.

Obesity is a strong risk factor in the development and progression of renal disease [3, 76, 77]. In the present study, FG-2216 reduced body weight, fat pad weight, and circulating cholesterol in Nx-Ob-ZSF1 rats. Other HIF-PHIs have shown similar improvements in obesity and lipid metabolism in mouse models of diabetes and metabolic dysfunction [52, 78, 79]. Moreover, decreased serum cholesterol correlated with reduced weight gain in LDL receptor–deficient mice treated with the HIF-PHI FG-4497 [80].

The reduced body and fat pad weights of Nx-Ob-ZSF1 rats treated with FG-2216 may be the result of caloric loss caused by the glycosuria observed in this treatment group. Increased urinary glucose excretion by FG-2216 could be related to reduced renal glucose reabsorption due to decreased expression of sodium/glucose cotransporters (SGLTs). In *in vitro* studies, HIF activation was associated with decreased expression of SGLTs in renal tubular cells [81]. *In vivo*, inhibiting SGLT2 improved glucose tolerance and increased urinary glucose excretion in rodent models of diabetes [82–84]. In animal and clinical studies, inhibiting SGLT2 improved GFR and reduced weight gain, kidney fibrosis, and mesangial expansion associated with type 2 diabetes [84–86].

FG-2216 did not improve fasting hyperglycemia or glucose utilization in Nx-Ob-ZSF1 rats during OGTTs performed throughout the study. This lack of effect on circulating blood glucose despite markedly increased urinary glucose excretion indicates that Nx-Ob-ZSF1 rats treated with FG-2216 for 14 weeks (the time of last OGTT) were still diabetic. A longer treatment duration with FG-2216 may be necessary to further reduce obesity and ameliorate glucose utilization in this model. This is in contrast to studies in other models of diabetes where HIF-PHIs improved glucose tolerance in mice fed a high fat diet and treated with FG-4497 for 4 weeks [78], and ameliorated insulin sensitivity in BTBR ob/ob mice treated for 18 weeks with enarodustat [52]. This difference between studies may be due to variation in tissue distribution of the different HIF-PHIs or to different animal models. The obese ZSF1 rat is a genetic model of diabetes that possesses two mutations of the leptin receptor [23] and it may prove more difficult to ameliorate glucose utilization with HIF-PHIs in this model.

HIFs may also affect weight gain and cholesterol by regulating lipid metabolism. In human fibroblasts and rodent models, HIF-1α induced the production of Insig-2 protein, which is involved in the degradation of HMG-CoA reductase, the rate limiting enzyme in cholesterol synthesis [87, 88]. Thus, HIF activation by PHIs may decrease hepatic levels of HMG-CoA reductase, thereby reducing cholesterol production. A similar mechanism may exist in humans. Roxadustat (FG-4592) reduced cholesterol in CKD patients with anemia during 16 to 19 weeks of treatment, and this effect was independent of treatments with statins or other lipid-lowering agents [89, 90].

Hypertension is common comorbidity of CKD [91, 92] seen in ZSF1 rats [23]. In our study, long-term administration of FG-2216 significantly reduced hypertension in Nx-Ob-ZSF1 rats. This finding is consistent with studies showing that HIF-PHIs reduce blood pressure. For example, Philipp et al. showed that FG-2216 reduced blood pressure in rats following aortocaval shunt surgery [20]. Flamme et al. showed that molidustat significantly lowered systolic blood pressure in a rat model of end-stage renal disease induced by 5/6 nephrectomy [17]. The mechanism underlying this effect on blood pressure may be due to HIF-induced changes in the transcription of genes involved in vasodilation under hypoxic conditions [93–95]. For example, HIF-1 upregulates genes that encode the hormone adrenomedullin, as well as the enzymes inducible nitric oxide synthase and heme oxygenase-1, which regulate production of the vasodilators nitric oxide and carbon monoxide, respectively [93, 96, 97]. In addition, increased urine sodium excretion observed in the later part of the study in Nx-Ob-ZSF1 rats treated with FG-2216 accompanied by elevated urine production in these animals may also have contributed to the correction of hypertension in this group.

Ob-ZSF1 rats develop heart failure characterized by abnormal diastolic function with increased stiffness of the left ventricle and prolonged relaxation time [28, 29, 39, 98]. These characteristics are associated with increased cardiac afterload and diabetic cardiac injury. The elevated afterload induces cardiac hypertrophy and fibrosis, and it increases end-systolic pressure and end-diastolic pressure. Treatment of Nx-Ob-ZSF1 rats with FG-2216 improved two secondary cardiac endpoints of the renal study: interstitial fibrosis and plasma concentration of the marker of cardiac hypertrophy NT pro-BNP. To confirm that FG-2216 had cardioprotective effects in addition to its renoprotective properties, we carried out a follow-up 16-week study with cardiac function as primary endpoint. In this study, WKY rats, a parental strain of ZFS1 rats, were used as the reference group instead of lean ZSF1 rats. Studies using both WKY and lean ZSF1 as controls to investigate heart failure in obese ZSF1 rats showed impaired hemodynamic parameters in lean ZSF1 rats compared to WKY such as LV dP/dt minimum, end-systolic pressure-volume relationship and heart rate [28, 29]. These results together with the elevated systemic blood pressure observed in lean ZSF1 rats [23, 39] led us to select WKY rats as the control strain for the cardiac function experiment. Treatment of Nx-Ob-ZSF1 rats with FG-2216 for 16 weeks reduced cardiac hypertrophy, and improved several diastolic function parameters, including EDP, EDV and IVRT compared with vehicle treatment.

In Ob-ZSF1 rats with intact kidneys, ejection fraction is initially preserved, but it declines as the animals age and develop late-stage heart failure [99]. In our study, Nx-Ob-ZSF1 rats with unilateral nephrectomy had reduced ejection fraction at 29 weeks of age compared to control WKY rats. This result was due to changes in end-diastolic volume because stroke volume was similar between control WKY and Nx-Ob-ZSF1 rats, whereas end-diastolic volume was increased in Nx-Ob-ZSF1 rats. FG-2216 improved ejection fraction by decreasing end-diastolic volume in Nx-Ob-ZSF1 rats while maintaining stroke volume. In previous work, FG-2216 improved cardiac hypertrophy and ejection fraction and reduced infarct size in mice and rats following myocardial infarction [21, 100]. FG-2216 also reduced cardiac dilation and improved ejection fraction in rats with heart failure induced by an aortocaval shunt [20]. In rats with aortocaval shunt, FG-2216 lowered end-diastolic pressure in the left ventricle, consistent with our findings. In the myocardial infarction model, FG-2216 improved cardiac function by increasing contractility in the left ventricle. In our study, FG-2216 did not affect contractility, as we did not observe any differences in dP/dt max or ESPVR between groups.

FG-2216 may indirectly improve cardiac function by reducing blood pressure, which decreases the load on the heart and the associated cardiac fibrosis and remodeling [101]. The cardioprotective effects of FG-2216 may also be mediated by increased production of the HIF-target gene EPO. The increased in erythropoiesis observed in Nx-Ob-ZSF1 rats treated with FG-2216 confirmed that the compound significantly stimulated EPO production in this model. Increases in circulating EPO levels 10 to 1000 folds over control levels have been reported in mice and monkeys after treatment with FG-2216 [18]. EPO has been shown to limit infarct size and left ventricle remodeling, and to improve cardiac function after myocardial infarction in rodents and pigs [102–105]. EPO also prevented pathological changes in rats with diabetic cardiomyopathy by increasing the number of peripheral blood endothelial progenitor cells [106]. In humans, treatment with EPO improved ejection fraction and clinical outcomes in patients with congestive heart failure [107, 108]. The putative mechanisms of EPO-induced cardioprotection have been linked to the anti-apoptotic, anti-inflammatory, and angiogenic effects of EPO [109–111].

Many studies have shown that HIF activation in pre-, post-, and remote-conditioning strategies improved cardiac outcomes after ischemia-reperfusion injury of the myocardium [32, 33]. Some of these mechanisms may also be cardioprotective in non-ischemic cardiomyopathies [33]. For example, HIF activation drives changes in gene expression that improve tissue perfusion and attenuate ischemic damage [93–95]. In our study, FG-2216 improved hypertension, suggesting that the compound vasodilates the systemic vasculature. Vasodilation of the coronary arteries may have increased cardiac blood flow and oxygenation of the myocardium, improving cardiac function. HIF activation can also reprogram cardiomyocyte metabolism from oxidative phosphorylation to anaerobic glycolysis. This switch decreases production of reactive oxygen species in mitochondria and reduces opening of mitochondrial permeability transition pores during ischemia-reperfusion [112]. Similar metabolic changes occurred in rats after treatment with the HIF-PHI GSK360A [113]. Long-term treatment with GSK360A (28 days) prevented the progressive reduction of ejection fraction and ventricular dilation in a rat model of ischemic heart failure [114]. In our study, FG-2216 decreased obesity and serum cholesterol in Nx-Ob-ZSF1 rats. Thus, metabolic reprogramming of cardiomyocytes may have occurred in these animals and contributed to their improved cardiac function.

## Limitations

In our studies, we accelerated disease progression by subjecting Ob-ZSF1 rats treated with FG-2216 or vehicle to unilateral nephrectomy at 8 weeks of age [24]. The non-obese reference groups were not nephrectomized to provide reference values in healthy animals for the different parameters measured in order to put in perspective the improvement in comorbidities achieved by FG-2216. The unilateral nephrectomy in the obese ZSF1 animals introduced an additional variable so the disease phenotype investigated in the study is the combination of obese ZSF1 comorbidities and unilateral nephrectomy. At study termination, GFR was lower in Nx-Ob-ZSF1 Vehicle rats than Ln-ZSF1 rats, but the reduction was not statistically different. Nx-Ob-ZSF1 rats displayed other indicators of kidney dysfunction, including increased proteinuria, kidney fibrosis, tubular damage, mesangial expansion, glomerulosclerosis, elevated serum creatinine, and elevated serum BUN. These results suggest that the Nx-Ob-ZSF1 rats in our study may have exhibited early stage renal failure with mildly decreased GFR. Further work evaluating later stages of renal failure would be valuable. Expression of the HIF target gene VEGF after treatment with FG-2216 was not measured in the present study. Therefore, a potential interaction between the HIF-PHI FG-2216 and the HIF-VEGF axis in relation to cardio-renal protection and diabetic macro- and microvascular disease was not addressed in this study using obese ZSF1 rats as a model of kidney failure with metabolic syndrome.

## Conclusions

Long-term intermittent treatment with the HIF-PHI FG-2216 improved nephropathy, cardiomyopathy, and obesity in Nx-Ob-ZSF1 rats, a model of kidney disease with metabolic syndrome. Our findings support that, in addition to correcting anemia, HIF-PHIs may exert reno- and cardioprotective effects in CKD patients with other comorbidities associated with kidney disease.

## Supporting information

S1 Fig FG-2216 did not affect serum BUN, serum creatinine or urine creatinine but increased urine sodium. Blood and urine were collected every four weeks during the treatment period to determine serum BUN (A), serum creatinine (B) urine creatinine (C) and urine sodium (D). Values represent mean ± SEM (n = 8–12). *P < 0.05 vs. Nx-Ob-ZSF1 Vehicle (Bonferroni test).
(TIF)

S2 Fig FG-2216 did not affect glucose tolerance in Nx-Ob-ZSF1 rats. Blood glucose levels during oral glucose tolerance tests after 10 weeks of treatment. Values represent mean ± SEM (n = 8–12). *P < 0.05 vs. Nx-Ob-ZSF1 Vehicle (Bonferroni test).
(TIF)

S3 Fig FG-2216 did not affect serum triglycerides. Blood was collected every four weeks during the treatment period to determine serum triglyceride levels. Values represent mean ± SEM (n = 8–12). *P < 0.05 vs. Nx-Ob-ZSF1 Vehicle (Bonferroni test).
(TIF)

S4 Fig FG-2216 had no effect on heart rate. Heart rate was measured at baseline and after 12 and 17 weeks of treatment. Values represent mean ± SEM (n = 8–12). *P < 0.05 vs. Nx-Ob-ZSF1 Vehicle (Bonferroni test).
(TIF)

S5 Fig FG-2216 had no effect on pulmonary water content. Lung tissue was harvested after 18 weeks of treatment and pulmonary water content was calculated. Values represent mean ± SEM (n = 8–12) (Dunnett’s test).
(TIF)

S6 Fig FG-2216 had no effect on cardiomyocyte size. Hearts were harvested after 18 weeks of treatment and stained with H&E. Circumference of cardiomyocytes was measured in the left ventricle (A) and septum (B). Values represent mean ± SEM (n = 9–15) (Dunnett’s test).
(TIF)

S1 Table Hematology parameters in Ln-ZSF1 and Ob-ZSF1 rats after 16 weeks of treatment. (DOCX)

S2 Table Parameters measured at the end of the cardiac study. (DOCX)
