# Supplementary figures and images for: A small-molecule inhibitor of hypoxia-inducible factor prolyl hydroxylase improves obesity, nephropathy and cardiomyopathy in obese ZSF1 rats
Source: PLoS One. 2021 Aug 2;16(8):e0255022. doi: 10.1371/journal.pone.0255022 (PMC8328318; doi:10.1371/journal.pone.0255022)

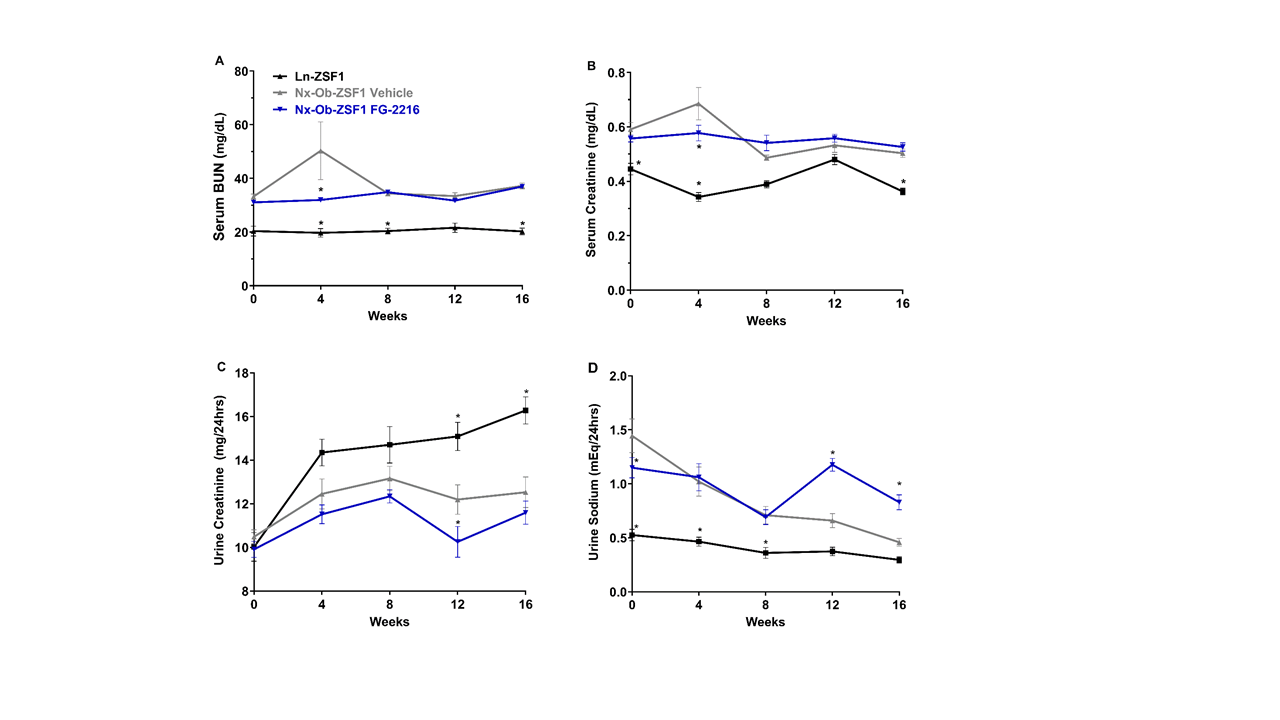

Supplement: S1 Fig — Blood and urine were collected every four weeks during the treatment period to determine serum BUN (A), serum creatinine (B) urine creatinine (C) and urine sodium (D). Values represent mean ± SEM (n = 8–12). *P < 0.05 vs. Nx-Ob-ZSF1 Vehicle (Bonferroni test). (TIF) [file pone.0255022.s001.tif]

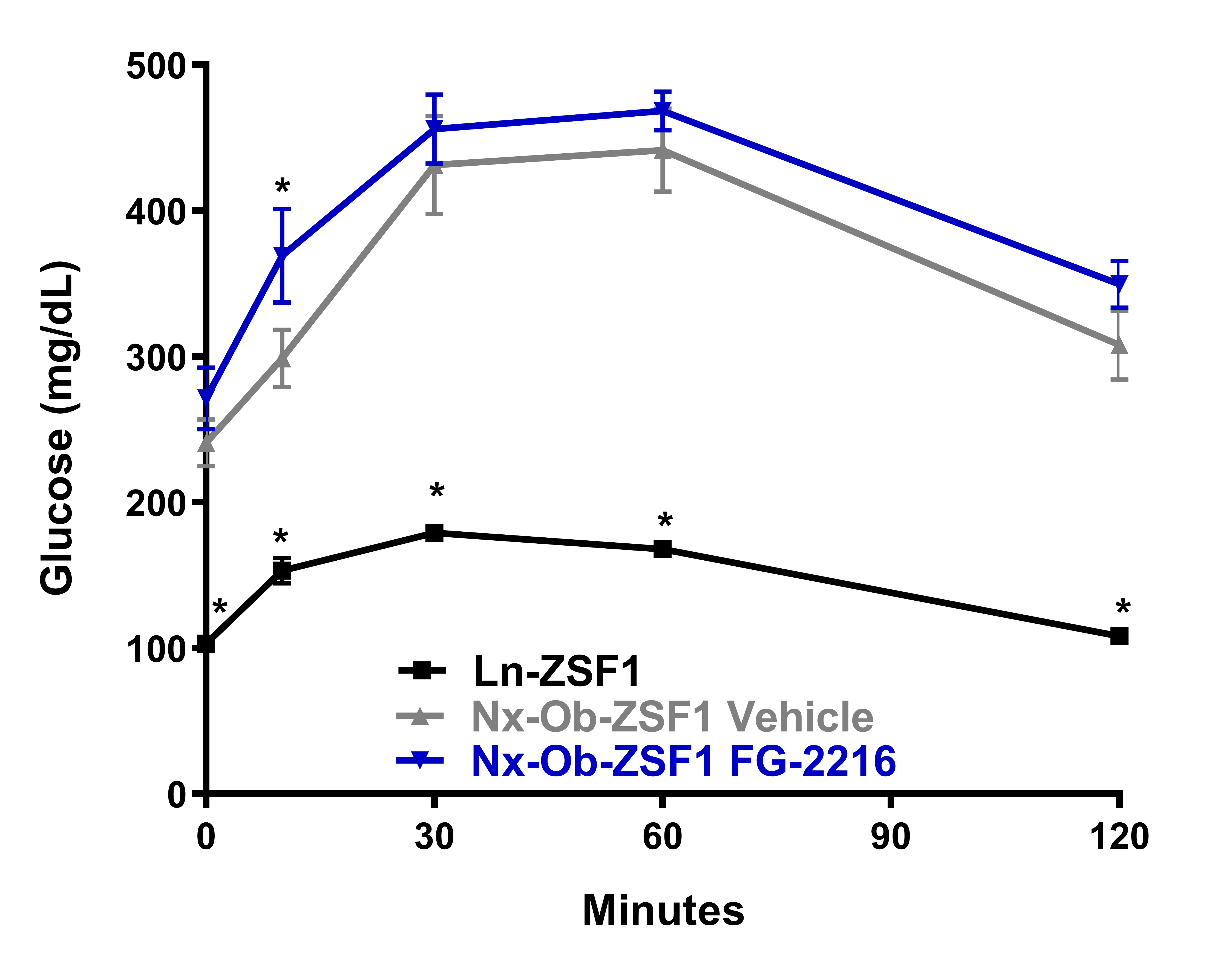

Supplement: S2 Fig — Blood glucose levels during oral glucose tolerance tests after 10 weeks of treatment. Values represent mean ± SEM (n = 8–12). *P < 0.05 vs. Nx-Ob-ZSF1 Vehicle (Bonferroni test). (TIF) [file pone.0255022.s002.tif]

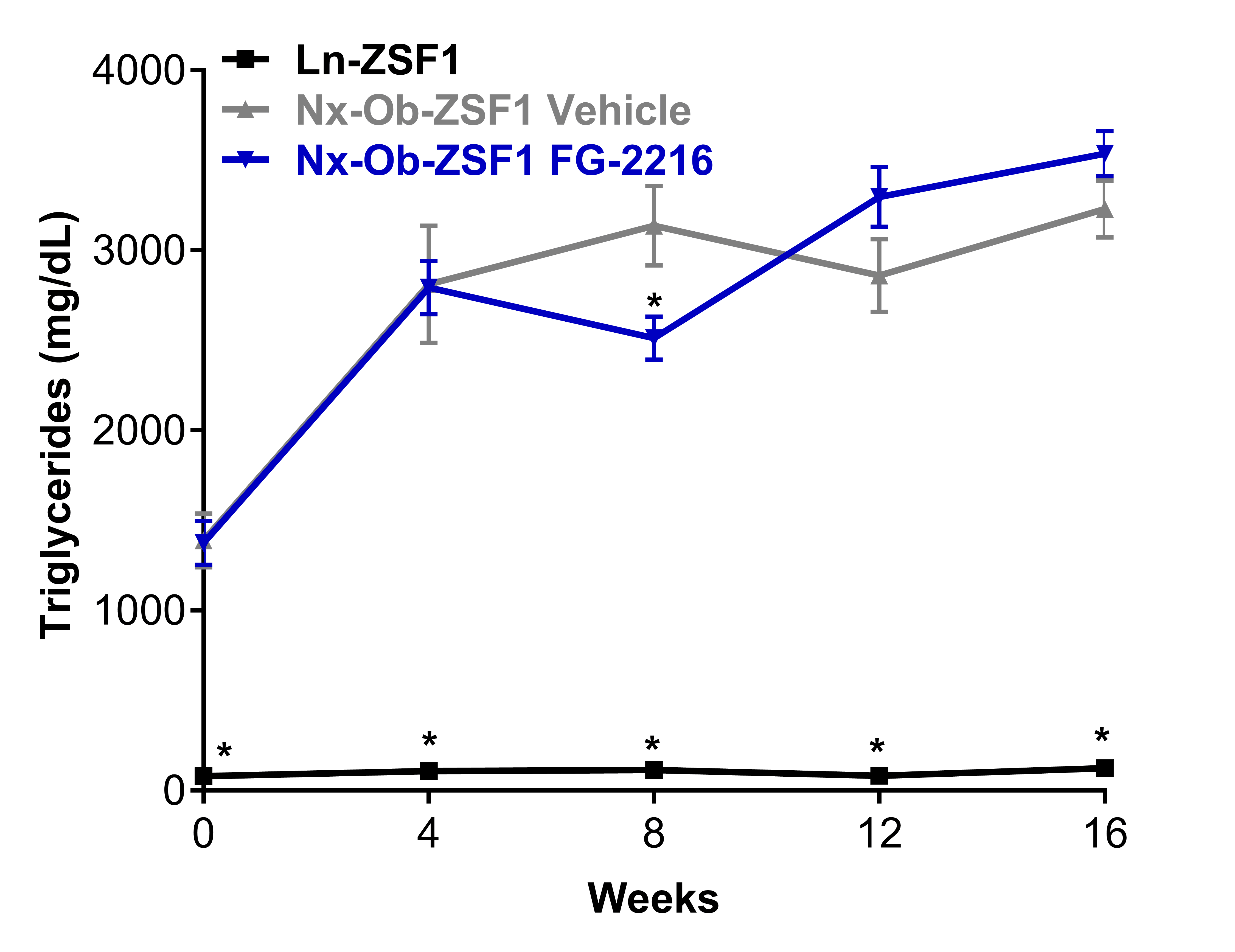

Supplement: S3 Fig — Blood was collected every four weeks during the treatment period to determine serum triglyceride levels. Values represent mean ± SEM (n = 8–12). *P < 0.05 vs. Nx-Ob-ZSF1 Vehicle (Bonferroni test). (TIF) [file pone.0255022.s003.tif]

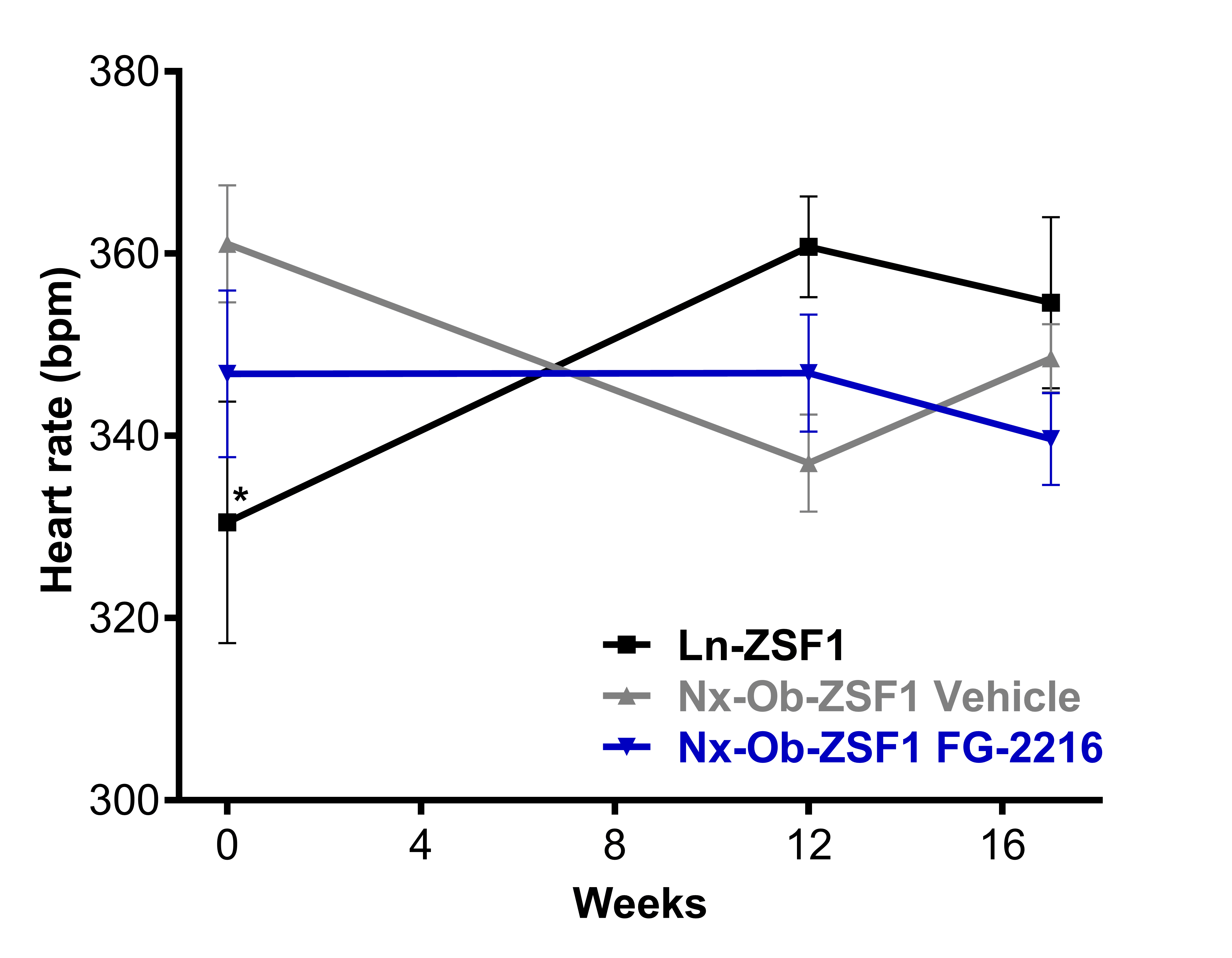

Supplement: S4 Fig — Heart rate was measured at baseline and after 12 and 17 weeks of treatment. Values represent mean ± SEM (n = 8–12). *P < 0.05 vs. Nx-Ob-ZSF1 Vehicle (Bonferroni test). (TIF) [file pone.0255022.s004.tif]

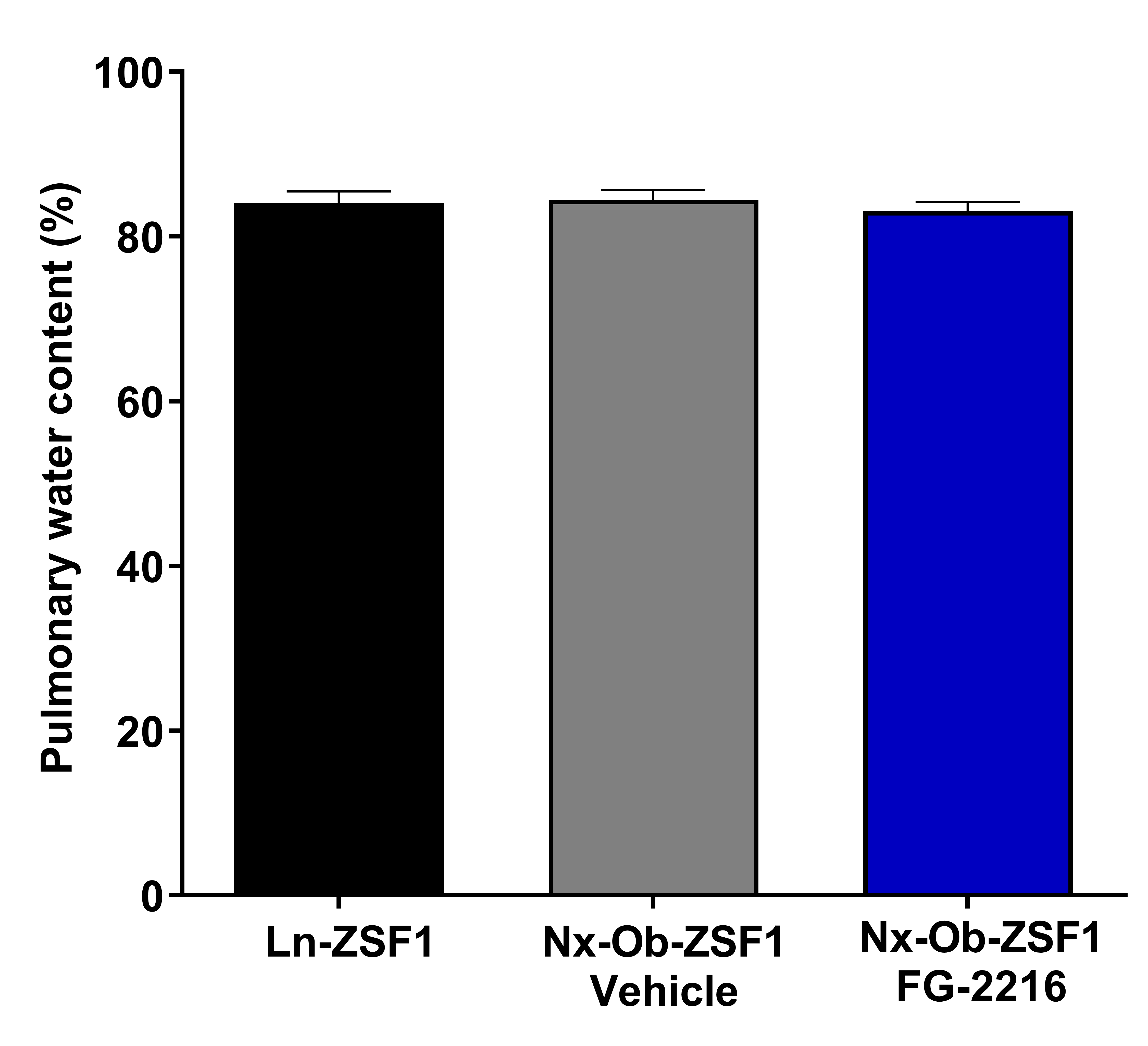

Supplement: S5 Fig — Lung tissue was harvested after 18 weeks of treatment and pulmonary water content was calculated. Values represent mean ± SEM (n = 8–12) (Dunnett’s test). (TIF) [file pone.0255022.s005.tif]

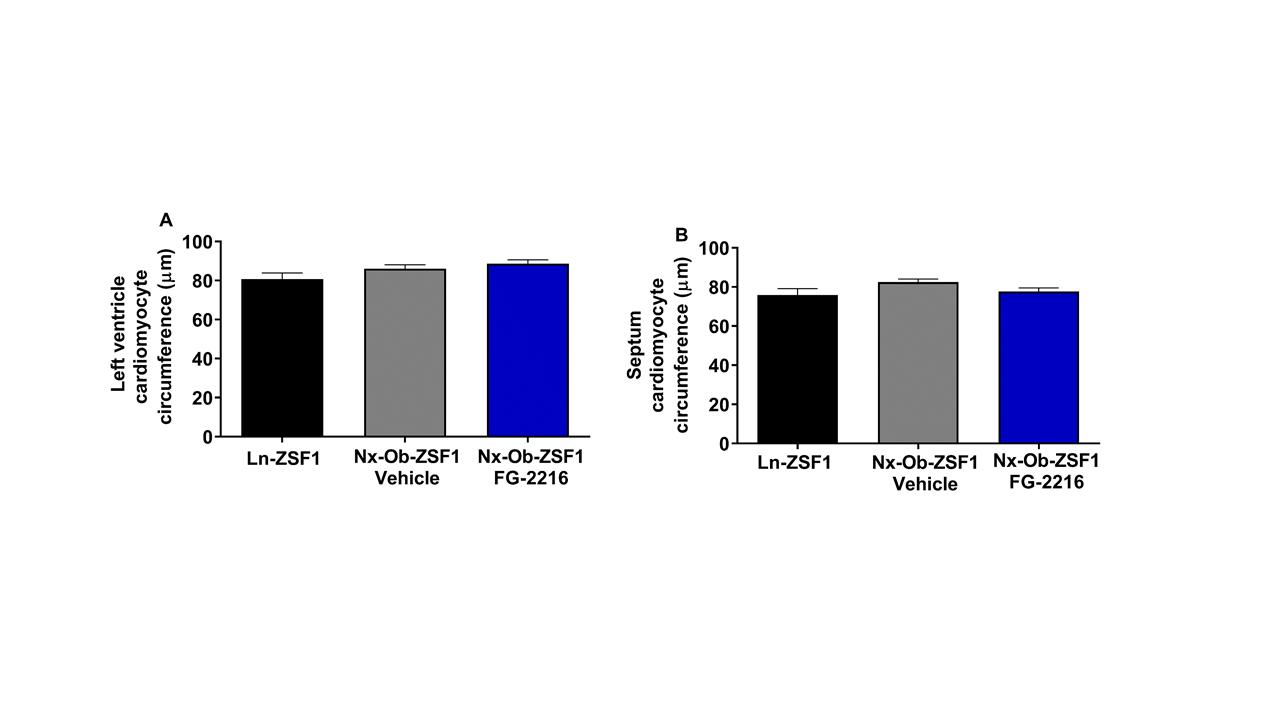

Supplement: S6 Fig — Hearts were harvested after 18 weeks of treatment and stained with H&E. Circumference of cardiomyocytes was measured in the left ventricle (A) and septum (B). Values represent mean ± SEM (n = 9–15) (Dunnett’s test). (TIF) [file pone.0255022.s006.tif]
