# Supplementary material for: A small-molecule inhibitor of hypoxia-inducible factor prolyl hydroxylase improves obesity, nephropathy and cardiomyopathy in obese ZSF1 rats
Source: PLoS One. 2021 Aug 2;16(8):e0255022. doi: 10.1371/journal.pone.0255022 (PMC8328318; doi:10.1371/journal.pone.0255022)
Supplement: S1 Table — (DOCX) [file pone.0255022.s007.docx]

**S1 Table. Hematology parameters in Ln-ZSF1 and Ob-ZSF1 rats after 16 weeks of treatment**

| Parameter | Ln-ZSF1 | Nx-Ob-ZSF1  Vehicle | Nx-Ob-ZSF1  FG-2216 |
| --- | --- | --- | --- |
| Hemoglobin (g/dL) | 16.2 ± 0.3* | 14.8 + 0.1 | 17.6 + 0.3* |
| Hematocrit (%) | 47.1 ± 1.0* | 40.7 ± 0.4 | 47.8 ± 0.9* |
| MCV (fL) | 48.3 ± 0.2 | 47.7 ± 0.3 | 52.6 ± 0.2* |
| MCH (pg) | 16.7 ± 0.1* | 17.4 + 0.1 | 19.3 + 0.1* |
| RBC (x10^6^/µL) | 9.8 ± 0.2* | 8.5 ± 0.1 | 9.1 ± 0.2 |
| MCHC (g/dL) | 34.4 ± 0.1* | 36.4 ± 0.3 | 36.8 ± 0.3 |

MCV=Mean Corpuscular Volume; MCH=Mean Corpuscular Hemoglobin; MCHC= Mean Corpuscular Hemoglobin Concentration; RBC=Red Blood Cell Counts

Mean ± SEM; n= 6 animals/Ln-ZSF1 group & n=10-12 animals/Nx-Ob-ZSF1 groups; **P* < 0.05 vs. Nx-Ob-ZSF1 Vehicle (Dunnett’s).
