# Supplementary material for: A small-molecule inhibitor of hypoxia-inducible factor prolyl hydroxylase improves obesity, nephropathy and cardiomyopathy in obese ZSF1 rats
Source: PLoS One. 2021 Aug 2;16(8):e0255022. doi: 10.1371/journal.pone.0255022 (PMC8328318; doi:10.1371/journal.pone.0255022)
Supplement: S2 Table — (DOCX) [file pone.0255022.s008.docx]

**S2 Table. Parameters measured at the end of the cardiac study**

|  | WKY | Nx-Ob-ZSF1  Vehicle | Nx-Ob-ZSF1  FG‑2216 |
| --- | --- | --- | --- |
| Hemoglobin (g/dL) | 18.6 ± 0.34* | 15.4 ± 0.25 | 18.3 ± 0.37* |
| Body weight (g) | 389 ± 6* | 694 ± 11 | 591 ± 17* |
| Fat pad weight | 5.56 ± 0.19* | 12.19 ± 0.48 | 10.17 ± 0.56* |
| Serum cholesterol (mg/dL) | 122 ± 2* | 377 ± 12 | 262 ± 10* |
| Heart weight (g) | 1.27 ± 0.04* | 1.72 ± 0.04 | 1.57 ± 0.06* |
| NT pro-BNP (pg/mL) | 72 ± 6* | 197 ± 18 | 119 ± 13* |
| Pulmonary water content (%) | 80.8 ± 0.7 | 80.2 ± 0.7 | 80.6 ± 0.4 |

Mean ± SEM; n=9 animals/WKY group; n=15 animals/Nx-Ob-ZSF1 Vehicle group; n=12 animals/Nx-Ob-ZSF1 FG-2216 group; **P* <0 .05 vs. Nx-Ob-ZSF1 Vehicle (Dunnett’s test).
